# Effects of Mind–Body Interventions Involving Meditative Movements on Quality of Life, Depressive Symptoms, Fear of Falling and Sleep Quality in Older Adults: A Systematic Review with Meta-Analysis

**DOI:** 10.3390/ijerph17186556

**Published:** 2020-09-09

**Authors:** Manuel Weber, Thiemo Schnorr, Mareike Morat, Tobias Morat, Lars Donath

**Affiliations:** 1 Institute of Movement and Sport Gerontology, German Sport University, 50933 Cologne, Germany; manuel.weber@stud.dshs-koeln.de (M.W.); thiemoschnorr@web.de (T.S.); t.morat@dshs-koeln.de (T.M.); 2 Institute of Exercise Science and Sport Informatics, German Sport University, 50933 Cologne, Germany; m.morat@dshs-koeln.de

**Keywords:** Tai Chi, Qigong, Yoga, Pilates, psychological health, psychological symptoms, prevention

## Abstract

*Background*: The aim of the present systematic meta-analytical review was to quantify the effects of different mind–body interventions (MBI) involving meditative movements on relevant psychological health outcomes (i.e., quality of life (QoL), depressive symptoms, fear of falling (FoF) and sleep quality) in older adults without mental disorders. *Methods*: A structured literature search was conducted in five databases (Ovid, PsycINFO, PubMed, SPORTDiscus, Web of Science). Inclusion criteria were: (i) the study was a (cluster) randomized controlled trial, (ii) the subjects were aged ≥59 years without mental illnesses, (iii) an intervention arm performing MBI compared to a non-exercise control group (e.g., wait-list or usual care), (iv) psychological health outcomes related to QoL, depressive symptoms, FoF or sleep quality were assessed and (v) a PEDro score of ≥5. The interventions of the included studies were sub-grouped into Tai Chi/Qigong (TCQ) and Yoga/Pilates (YP). Statistical analyses were conducted using a random-effects inverse-variance model. *Results*: Thirty-seven randomized controlled trials (RCTs) (comprising 3224 participants) were included. Small to moderate-but-significant overall effect sizes favoring experimental groups (Hedges’ *g*: 0.25 to 0.71) compared to non-exercise control groups were observed in all outcomes (all *p* values ≤ 0.007), apart from one subdomain of quality of life (i.e., social functioning, *p* = 0.15). Interestingly, a significant larger effect on QoL and depressive symptoms with increasing training frequency was found for TCQ (*p* = 0.03; *p* = 0.004). *Conclusions*: MBI involving meditative movements may serve as a promising opportunity to improve psychological health domains such as QoL, depressive symptoms, FoF and sleep quality in older adults. Hence, these forms of exercise may represent potential preventive measures regarding the increase of late-life mental disorders, which need to be further confirmed by future research.

## 1. Introduction

Adults aged 60 years and older are nowadays the fastest growing segment of the population, whereas at the same time the prevalence of mental illnesses in older adults is rising [1,2]. Psychological outcomes can be improved by regular physical activity and exercise in adults of all ages [3]. The fact that psychological symptoms are commonly experienced in later life reveals a need for age-appropriate exercise programs as preventive measures that do not only address physical but also psychological health in older people [4,5]. A potential approach is offered by alternative forms of exercise (e.g., mind–body interventions (MBI)) with low impact, low demands on the muscles and less energy expenditure [4]. Mind–body interventions involving meditative movements include, but are not limited to, Tai Chi, Qigong, Yoga, Pilates and Feldenkrais. These forms of exercise use movement in conjunction with meditative attention [6]. There are active and passive breathing-based components that are characterized by a variety of actions such as stretching and relaxation of skeletal muscles, coordinated body, high levels of concentration, regular breathing movements and breathing techniques [7,8]. Furthermore, MBI involving meditative movements are mostly offered in supervised group training sessions containing the potential for psychosocial benefits. Another great advantage is that they can easily be adjusted to a person’s individual performance level, which forms an essential requirement when focusing on older adults. The underlying multifactorial approach of MBI involving meditative movements is believed to directly affect health by interactions between physical, mental, social and behavioral factors [9,10]. Since these types of exercise did not experience increasing interest in western civilizations before the last decade, they have not been thoroughly investigated. Nevertheless, MBI involving meditative movements already showed that they are suitable for the special needs of older adults as a target group [6,11]. That’s why the latest findings considered MBI involving meditative movements as being feasible and effective for all ages [11,12,13,14,15]. Furthermore, MBI involving meditative movements attracted researchers’ attention because of their effectiveness in treating depression and various chronic illnesses, including stress, acute and chronic pain, cardiovascular disease and hypertension in the adult population [16,17]. A tendency towards beneficial effects on quality of life in older people can be observed, since psychological well-being may be ameliorated and anxiety may be decreased [18]. However, existing reviews especially on Qigong, Yoga and Pilates for older adults are limited and do not provide firm conclusions on its effectiveness on quality of life, depression and self-efficacy [19].

Depression represents a significant psychological health problem worldwide. Older people are particularly vulnerable to depression [20]. Moreover, old-age depression contributes to deterioration in functioning, an exacerbation of medical conditions and increased healthcare costs. The first step to slowing or preventing the development of depression is controlling depressive symptoms [21,22]. As older adults often have lower physical-function capacity than younger adults, forms of exercise suitable for older people are required [21]. There is evidence that MBI have short-term effects in alleviating depressive symptoms among older adults [22], but systematic reviews and meta-analyses comparing several MBI involving meditative movements are lacking.

One-third of older people over 65 years fall once or more annually [23]. Falls result in disability, injury, hospitalization and loss of independence in older people. Associations between fear of falling and reductions in physical and social activities as well as negative impacts on quality of life exist [24]. The risk of future falls can be increased by high levels of fear of falling, while low levels can be protective for falling, regardless of the presence of balance impairments [25]. The favorable effects of Tai Chi and Qigong on reducing fear of falling in older adults are documented in various reviews [26,27,28], but they have been published several years ago having concluded that more high-quality trials were needed to strengthen the evidence base. Moreover, there is a lack of systematic reviews with meta-analysis regarding the effects of Yoga and Pilates on fear of falling.

Another major public health problem for the elderly are sleep disorders. Significant negative impacts on psychological and physical health, such as impairments in the metabolic, endocrine and immune systems leading to daytime function impairment, mood disturbances and reduced quality of life can be caused by sleep disorders [29]. Poor sleep quality may even be a risk for the onset of Alzheimer’s disease [30]. In the adult population, MBI involving meditative movements have already demonstrated to improve self-reported sleep quality and may even have similar effects in older people with sleep complaints [29,31].

The older population is more likely to be on medicines for different medical conditions. Sometimes, the medical conditions and the medications themselves may notably affect quality of life, mood and sleep. MBI involving meditative movements are required to be studied in older people as they are not commonly associated with side effects or drug–drug interactions that are generally more common in this age group [6]. However, a paucity of evidence exists on the mental health benefits of non-traditional modes of exercise, such as MBI involving meditative movements [32]. Recent reviews and meta-analyses are mostly limited to cognitive performance [13,14] and physical function [11,15], above all among older adults. The existing literature usually examined psychological health outcomes not primarily, but secondarily or tertiarily, and still showed promising results, for example, positive effects on self-awareness of the body and hence an increase of mental clarity and the ability of an individual to tolerate physical discomfort [33]. Furthermore, the majority of those focusing on older people were not restricted to randomized controlled trials (RCTs) and high methodological quality and generally focused on only one type of intervention. Besides, new evidence from additional randomized controlled trials has become available. Facing the increasing prevalence of mental disorders in older adults, non-pharmacological approaches as preventive measures are wanted for reducing psychological symptoms (i.e., depressive symptoms, fear of falling, poor sleep quality) since these symptoms can severely diminish quality of life [22,32,34]. Maintenance of quality of life is considered one the most fundamental outcomes of care services for older adults [35]. The importance of quality of life is endorsed by several international action plans on ageing and the measurement of quality of life among older adults shows growing international interest [35,36,37].

Based on the outlined state of research, quality of life, depressive symptoms, fear of falling and sleep quality represent important indicators of psychological health among older adults with the result that the purposes of the current review and meta-analysis were: (a) to summarize the scientific literature on MBI involving meditative movements and compare its effects versus non-exercise control groups; (b) to quantify the effect sizes of adaptations in several relevant psychological health outcomes (i.e., quality of life, depressive symptoms, fear of falling and sleep quality); (c) to perform subgroup analyses in order to evaluate potential moderating effects of intervention type and frequency; and (d) to draw conclusions on the potential value of MBI involving meditative movements concerning the prevention of late-life mental disorders by reducing psychological symptoms.

## 2. Materials and Methods

### 2.1. Search Strategy

The current meta-analysis was performed and reported in accordance with the PRISMA guidelines [38]. A systematic literature search was conducted independently by two researchers (M.W., T.S.) until the 20 December 2019 in the following biomedical and psychological databases: Ovid, PsycINFO, PubMed, SPORTDiscus and Web of Science. Relevant search terms or operators were combined with Boolean operators (OR/AND) and were applied on three search levels (Table 1).

### 2.2. Eligibility Criteria

The subsequent inclusion criteria based on the PICOS approach (population [P], intervention [I], comparators [C], outcomes [O], and study design [S]) [38] were used:

- Full-text articles published in English in a peer-reviewed journal;
- Participants were older adults aged 59 years or above and the mean age of study samples was ≥65 years (P);
- Mind–body interventions involving meditative movements as a generic term for interventions at low to moderate intensity uniting a meditative state of mind, conscious breath and movement such as Tai Chi or Yoga (I);
- Control groups that did not follow physical exercise intervention like aerobic exercise, strength exercise, any mind–body intervention, stretching, or relaxation exercise (CON) as comparators (e.g., wait-list control group, inactive control group, health education or usual care) (C);
- Parameters related to quality of life, depressive symptoms, fear of falling and sleep quality served as outcome measures (O);
- (Cluster) randomized controlled intervention studies with pre- and post-testing (S);
- A PEDro (Physiotherapy Evidence Database) score of at least five to achieve high methodological quality.

The exclusion criteria were:

- Older adults with mental disorders such as mild cognitive impairment (MCI), dementia or depression;
- No adequate control condition or control group;
- No regular supervised intervention during the study period;
- Intervention not delivered in group format;
- Intervention without movement (e.g., guided imagery);
- Outcome not assignable to target outcomes (see inclusion criteria).

### 2.3. Assessment of Methodological Study Quality

Study quality was assessed using the PEDro (Physiotherapy Evidence Database) scale [39]. The PEDro scale contains 11 dichotomous items (yes or no) to characterize randomization, internal and external validity and statistical information of the respective trials of interest, which were rated independently by two researchers (M.W., T.S.). Criterion 1 is not used to calculate the PEDro score even though it relates to the external validity. Both non-blinded researchers must have obtained consensus on every item. To achieve final consensus, in case of disagreement between both researchers, a third independent researcher (L.D.) was consulted. If the total PEDro score was lower than five, the study was excluded since studies with low methodological quality can lead to biased results.

### 2.4. Data Extraction and Interventions

Two investigators (M.W., T.S.) independently performed data extraction. The following data were extracted and transferred to an excel spread sheet: author(s), year, study design, sample (number and age of the participants), intervention characteristics (duration, frequency, type, intensity) as well as the results of the outcome measures (pre- and post-test means and standard deviations). Main outcome parameters were the mean changes in target outcomes from baseline to post-intervention measures in the exercise groups compared to non-exercise control groups. If the exact pre- and post-test values were not available in a study, requests to the authors for data were made via email. After having sent a second reminder and still not having received any answer, the records were excluded.

If included studies contained more than one measuring tool for assessing the same target outcome, the effect size and standard error were pooled together. Since the dimension ‘quality of life’ represents a complex construct with several subdomains, three of them called physical, psychological and social functioning were split off and investigated separately apart from the overall quality of life for a more detailed analysis. Therefore, all used questionnaires related to quality of life with individual subscales of which the scores were indicated, were allocated to the above-named subdimensions. A questionnaire which did not allow allocation due to missing values of subscales or missing subscales at all implied exclusion from calculations. An overview of the classification made by the authors is shown in Table 2. The values of subscales that could not be clearly assigned to one of the three subdimensions were ignored and thus are not listed in Table 2.

The present review contained mind–body interventions involving meditative movements. At the end of study selection process, the performed interventions in all appropriate studies included one of the following: Tai Chi, Qigong, Yoga or Pilates. Tai Chi and Qigong were grouped together. They were considered equivalent interventions, following other recent reviews [9,40], and because they are closely related having their origin in the martial art of China [29]. The other subgroup consisted of Yoga and Pilates underlying lots of similarities [41]. Additionally, the description of Yoga and Pilates interventions of the included studies revealed comparable contents.

### 2.5. Statistical Analysis

Standardized mean differences (SMD, with 90% confidence intervals, CI) from pre- to post-test for each variable and for each study arm were computed using the adjusted Hedges’ *g* (Equation (1)). The data of the relevant subscales regarding quality of life were pooled together by forming the mean of the effect sizes and standard errors. According to Deeks and Higgins [42] the inverse-variance method was conducted using the random effects model [43]. The Cochrane Review Manager Software (RevMan 5.3.5, Cochrane Collaboration, Oxford, UK) was used to compute statistical analyses. Forest plots with 90% CI were generated for each outcome category (quality of life (overall, physical, psychological and social functioning), depressive symptoms, fear of falling and sleep quality). The magnitude of SMD was classified according to the following scale: 0–0.19 = negligible effect, 0.20–0.49 = small effect, 0.50–0.79 = moderate effect and ≥0.80 = large effect [44]. A funnel plot evaluation was performed to examine a potential publication bias.

(1) $${SMD}_{i}\;=\;\frac{m_{1i}{-m}_{2i}}{s_{i}}\left(1-\frac{3}{{4N}_{i}-9}\right)$$

## 3. Results

### 3.1. Trial Flow

**Figure 1:**
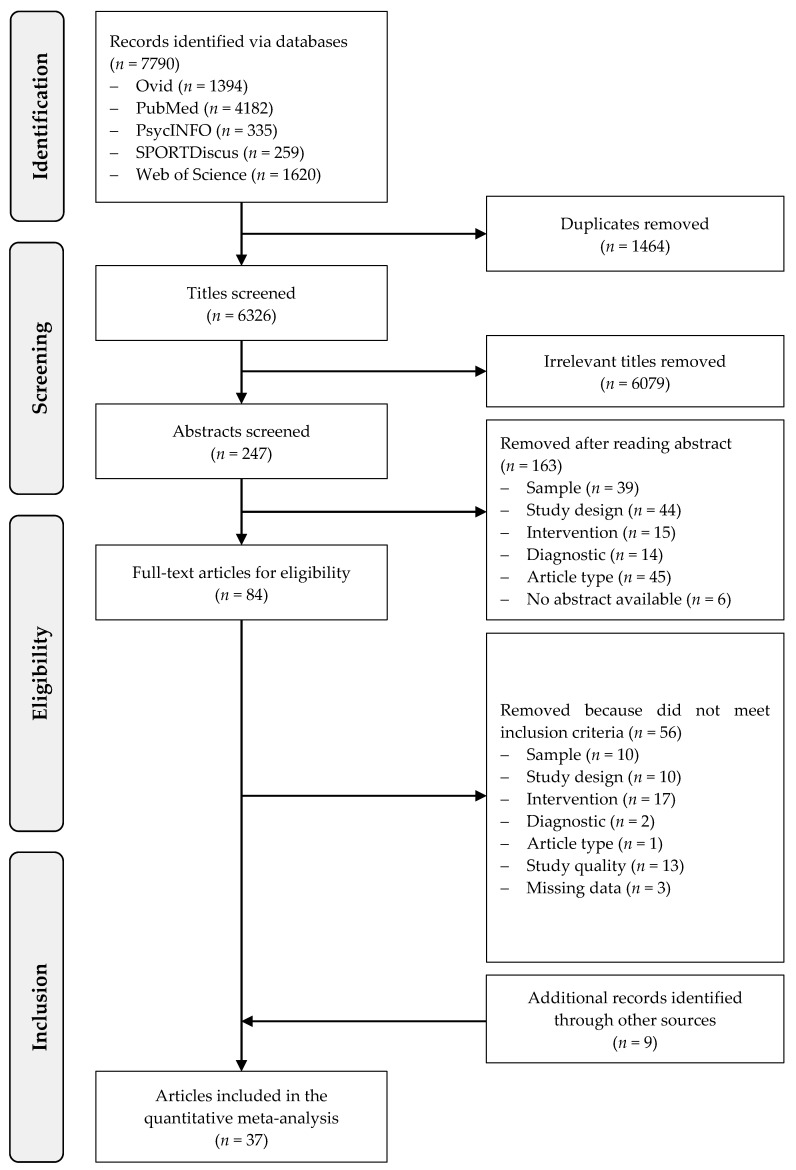
Flow of study screening and selection.

Throughout the search procedure 7790 articles were identified as potentially relevant (Figure 1). After having removed duplicates and screened titles, 247 abstracts and 84 full texts were carefully screened of which 28 met the inclusion criteria. The exclusion category ‘diagnostic’ meant that the outcomes and/or instruments were not adequate and the category ‘missing data’ referred to a lack of information despite contacting the authors. Through other reviews and sources nine additional articles were identified. Hence, 37 articles were included in final quantitative meta-analysis. The country where most of the studies (10 out of 37) were conducted is China, followed by the USA (8 studies) and Iran (5 studies). All countries are listed in Table S1 in the Supplementary Materials.

### 3.2. Study Characteristics

#### 3.2.1. Participants

In total, 3224 participants aged 59 years and above (72.2 ± 7.3) were involved in the included 37 randomized controlled trials [45,46,47,48,49,50,51,52,53,54,55,56,57,58,59,60,61,62,63,64,65,66,67,68,69,70,71,72,73,74,75,76,77,78,79,80,81] (Table S1). The mean sample size was 93 ± 56.7 subjects ranging from 17 [74] to 269 [60]. Community-dwelling older adults were investigated by most studies (32 out of 37), whereas one study included both community-dwelling older adults and inhabitants of retirement centers [70]. The other ones consisted of residents of elderly homes [51,80], long-term care facilities [53,54] or a retirement center [69]. Seven studies analyzed individuals with chronic physical conditions, namely the following: older women with senile osteoporosis [57], older adults with chronic obstructive pulmonary disease (COPD) [68], postmenopausal women with osteopenia [78], older adults with hip or knee osteoarthritis [47], older adults with hyperkyphosis (noticed after age 50) [49], older adults with knee osteoarthritis [55] and older adults with chronic low back pain [70]. One study involved older adults with moderate sleep complaints [56]. Six studies exclusively included women [45,46,57,61,78,79] and one study only men [69].

#### 3.2.2. Interventions

Eight studies were three-arm and 29 studies two-arm RCTs. In three cases, all arms were included in the meta-analysis since two arms were mind–body interventions involving meditative movements [65,70,79]. All appropriate studies involved either Tai Chi, Qigong, Yoga or Pilates. The most frequent mind–body intervention was Tai Chi [47,51,52,53,54,55,56,57,58,59,60,61,62,63,65,66,67,69,75,78,80]. Mainly, the Tai Chi Yang style (24 forms or 10 simplified forms) was applied. Yoga was performed in 10 studies [49,50,64,65,67,70,71,72,74,77], whereas Qigong appeared in five study arms [70,73,79,81], Pilates three times [45,46,48] and Tai Chi Qigong only twice [68,76]. Each mind–body intervention was an isolated treatment given to the intervention group, except for one study that combined Tai Chi with cognitive behavioral strategies [55]. Oftentimes, the intensity was not mentioned, but if it was, low intensity and very seldomly moderate intensity was stated. Unfortunately, this information was not further specified. The length of the intervention varied between four weeks [74] and one year [59] with a mean length of 15.2 weeks (*SD* = 8.7). In three studies, one exercise session per week took place [67,70,71] while in other three studies the subjects even exercised five times a week [55,66,81]. However, the majority of the studies followed the frequency two, respectively, three exercise units per week (2.6 ± 1.0). The attendance rates for the experimental groups, where indicated, varied from 61% [47] to 96% [65]. The following non-exercise control groups were used by the included trials: no treatment/normal lifestyle/activities of daily living [48,51,52,55,57,62,63,64,66,69,72,75,76,78,79,80], wait-list [47,58,67,70,71,77,81], usual/standard care [53,54,60,68], health education [50,56,59,61], series of guidelines [45,46], monthly lunch/seminars [49], telephone calls [65], newspaper reading group [73], socialization group [74]. More detailed information is shown in Table S1.

#### 3.2.3. Outcomes and Instruments

The investigated outcomes of the included studies were quality of life—including three derived subscales: physical, psychological and social functioning—depressive symptoms, fear of falling and sleep quality. Quality of life was examined the most (21 out of 37 studies), whereby various versions of the Short Form Health Survey (SF-36/12/20) were the most popular measuring instrument. Depressive symptoms were assessed in 17 studies, whereas seven different questionnaires were applied. Fear of falling was involved in 11 trials and mainly assessed by modified versions of the Falls Efficacy Scale (FES/MFES/FES-I/short FES-I) or the Activities-Specific Balance Confidence Scale (ABC). By contrast, sleep quality, which was always assessed by the Pittsburgh Sleep Quality Index (PSQI), turned out to be the least represented dimension (eight studies). All used questionnaires are summarized in Table S2.

### 3.3. Risk of Bias Assessment

The funnel plot evaluation showed no evidence of asymmetry and publication bias. The funnel plots of all different outcome measures are shown in Figures S1 and S2 in the Supplementary Materials. The mean of the study quality (PEDro score) was 6.5 (median of 6.0; *SD* = 1.1) with a range between five and eight (Table S3). Only 9 out of 37 studies had a PEDro score of five [51,58,63,64,73,74,78,79,80]. None of the studies blinded the subjects or therapists, which is considered to be generally difficult within exercise intervention studies. Two studies showed a dropout rate of more than 15% [59,68]. Half of the trials (19 out of 37) did not conduct an intention-to-treat analysis (Table S3). Nevertheless, items 1, 2, 4, 10 and 11 were fully complied by all included studies.

### 3.4. Effects on Quality of Life

#### 3.4.1. Overall

**Figure 2:**
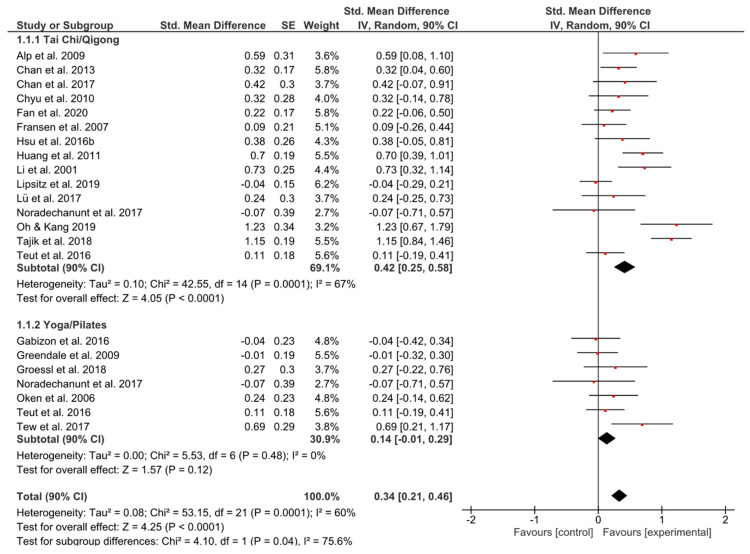
Standardized mean effects of mind–body interventions on quality of life compared to a non-exercise control group. Data are separately presented for Tai Chi/Qigong and Yoga/Pilates. SE: standard error; IV: inverse variance model; CI: confidence interval; Std.: standardized.

The results of the meta-analysis on overall quality of life showed in the Tai Chi/Qigong (TCQ) group as well as in the Yoga/Pilates (YP) group small but relevant effects (*g* = 0.42, 90% CI [0.25, 0.58]; *p* = 0.0001; *g* = 0.14, 90% CI [−0.01, 0.29]; *p* = 0.12) (Figure 2). The total effect was significant favoring the experimental group (*g* = 0.34, 90% CI [0.21, 0.46]; *p* < 0.0001). The overall *I^2^* indicated that 60% of the variability across studies was due to heterogeneity rather than chance.

#### 3.4.2. Physical Functioning

**Figure 3:**
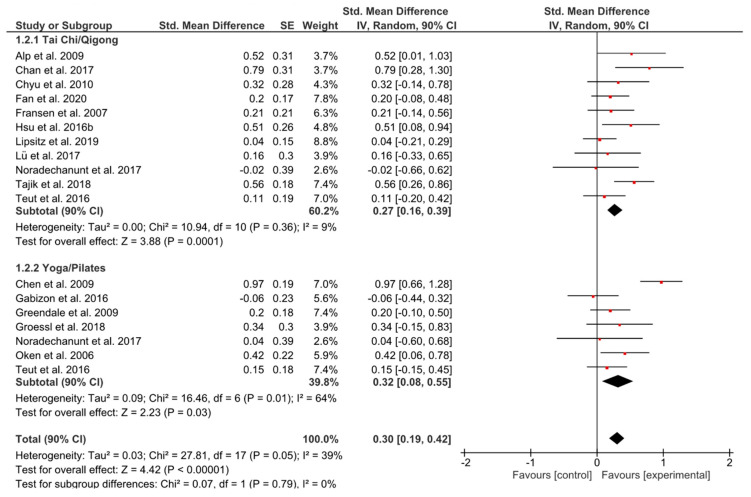
Standardized mean effects of mind–body interventions on physical functioning (subscale of quality of life) compared to a non-exercise control group. Data are separately presented for Tai Chi/Qigong and Yoga/Pilates. SE: standard error; IV: inverse variance model; CI: confidence interval; Std.: standardized.

Both groups presented similar small but significant effect sizes (*g* = 0.27, 90% CI [0.16, 0.39]; *p* = 0.0001; *g* = 0.32, 90% CI [0.08, 0.55]; *p* = 0.03), whereas there were no subgroup differences (*p* = 0.79) (Figure 3).

#### 3.4.3. Psychological Functioning

**Figure 4:**
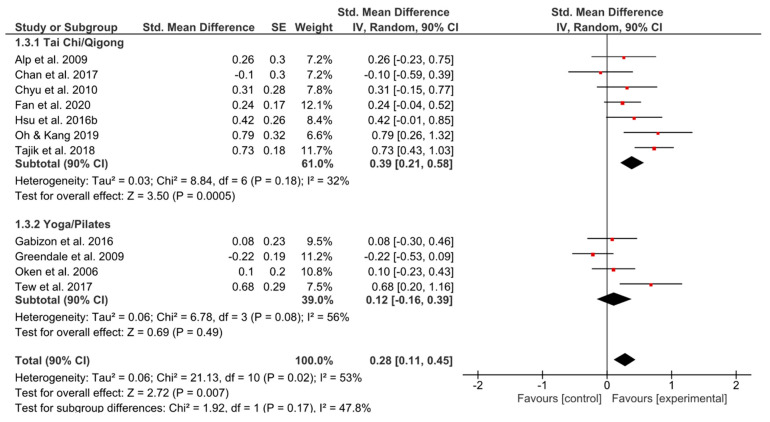
Standardized mean effects of mind–body interventions on psychological functioning (subscale of quality of life) compared to a non-exercise control group. Data are separately presented for Tai Chi/Qigong and Yoga/Pilates. SE: standard error; IV: inverse variance model; CI: confidence interval; Std.: standardized.

Only TCQ showed a significant effect (*g* = 0.39, 90% CI [0.21, 0.58]; *p* = 0.0005) and simultaneously small heterogeneity (*I^2^* = 32%) (Figure 4).

#### 3.4.4. Social Functioning

**Figure 5:**
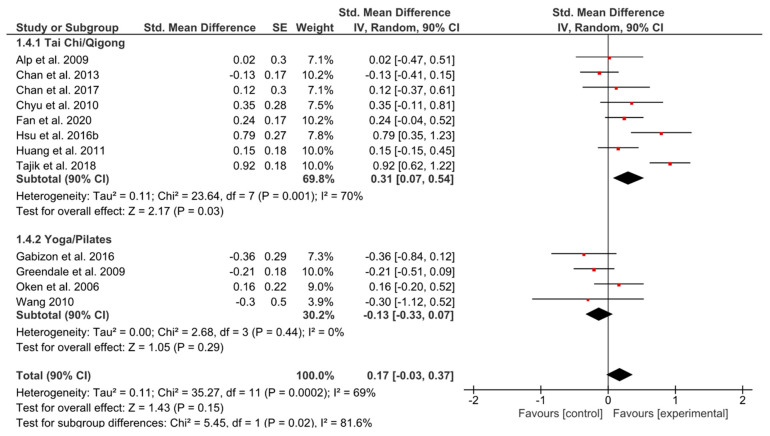
Standardized mean effects of mind–body interventions on social functioning (subscale of quality of life) compared to a non-exercise control group. Data are separately presented for Tai Chi/Qigong and Yoga/Pilates. SE: standard error; IV: inverse variance model; CI: confidence interval; Std.: standardized.

Even though the effect of YP favored the control group with a small heterogeneity, the effect was non-significant (*g* = −0.13, 90% CI [−0.33, 0.07]; *p* = 0.29; *I^2^* = 0%) (Figure 5). By contrast, a positive significant effect (*g* = 0.31, 90% CI [0.07, 0.54]; *p* = 0.03) could be seen for TCQ.

### 3.5. Effects on Depressive Symptoms, Fear of Falling and Sleep Quality

#### 3.5.1. Depressive Symptoms

**Figure 6:**
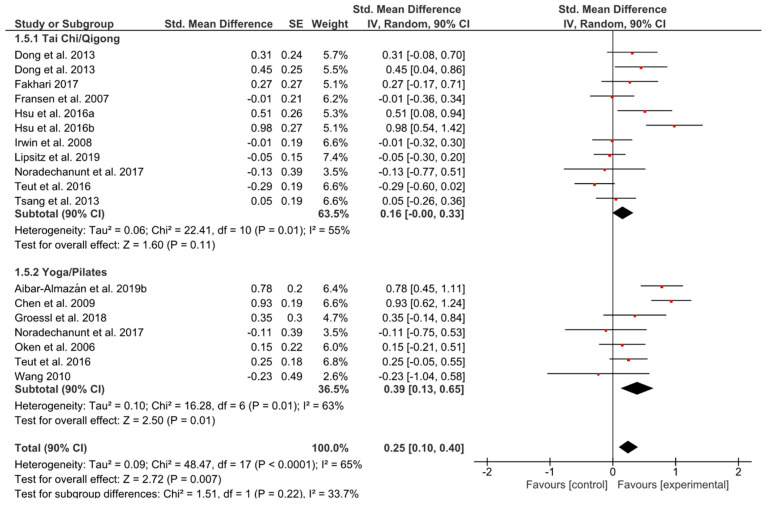
Standardized mean effects of mind–body interventions on depressive symptoms compared to a non-exercise control group. Data are separately presented for Tai Chi/Qigong and Yoga/Pilates. SE: standard error; IV: inverse variance model; CI: confidence interval; Std.: standardized.

A small and significant overall effect could be observed (*g* = 0.25, 90% CI [0.10, 0.40]; *p* = 0.007) (Figure 6). The heterogeneity in both groups could be considered high (*I^2^* = 55%; *I^2^* = 63%). YP seemed to induce higher effects compared to TCQ (*g* = 0.39, 90% CI [0.13, 0.65]; *p* = 0.01 vs. *g* = 0.16, 90% CI [0.00, 0.33]; *p* = 0.11).

#### 3.5.2. Fear of Falling

**Figure 7:**
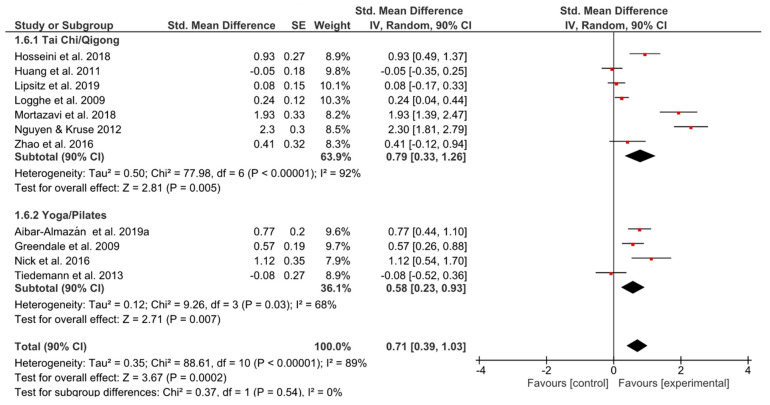
Standardized mean effects of mind–body interventions on fear of falling compared to a non-exercise control group. Data are separately presented for Tai Chi/Qigong and Yoga/Pilates. SE: standard error; IV: inverse variance model; CI: confidence interval; Std.: standardized.

The most noticeable effect sizes of the whole meta-analysis were found in the outcome ‘fear of falling’ (TCQ: *g* = 0.79, 90% CI [0.33, 1.26]; *p* = 0.005; YP: *g* = 0.58 [0.23, 0.93]; *p* = 0.007) (Figure 7). Nevertheless, the width of the 90% confidence intervals suggested a large amount of inconsistency among effect sizes.

#### 3.5.3. Sleep Quality

**Figure 8:**
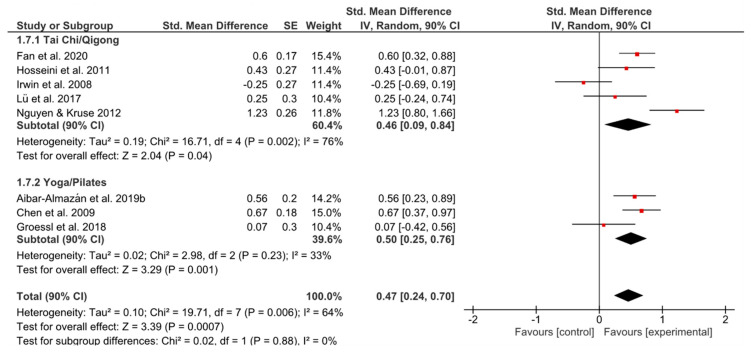
Standardized mean effects of mind–body interventions on sleep quality compared to a non-exercise control group. Data are separately presented for Tai Chi/Qigong and Yoga/Pilates. SE: standard error; IV: inverse variance model; CI: confidence interval; Std.: standardized.

Both groups showed a comparably moderate effect size (Figure 8). Heterogeneity of YP was lower (*I^2^* = 33% vs. *I^2^* = 76%), but the analysis contained three studies only. The overall effect favored the experimental group having a significant effect of *g* = 0.47 (90% CI [0.24, 0.70]; *p* = 0.0007).

### 3.6. Effects of Training Frequency

For both subgroups, an additional analysis regarding exercise frequency was conducted. The cut-off point was set between two and three sessions per week. There was a significant higher effect on overall quality of life for TCQ that exercised three times or more a week comparing to one or two times a week (*g* = 0.60, 90% CI [0.36, 0.84] vs. *g* = 0.20, 90% CI [0.04, 0.37]; *p =* 0.03). The same significant difference was shown in the variable ‘depressive symptoms’ (*g* = 0.39, 90% CI [0.16, 0.62] vs. *g* = −0.08, 90% CI [−0.22, 0.07]; *p* = 0.004). No significant difference (*p* = 0.88) was found in fear of falling comparing three and more sessions per week (*g* = 0.84, 90% CI [0.21, 1.48]) with fewer sessions per week (*g* = 0.74, 90% CI [−0.22, 1.71]). The results of the other outcomes and YP were not representative on account of too few studies and thus not further specified.

## 4. Discussion

Psychological health parameters have frequently been recorded as secondary or tertiary outcomes when investigating the effects of mind–body interventions (MBI) involving meditative movements in older adults. Although positive effects of several forms of MBI on global well-being have been demonstrated [18], the significance of preventive effects among older adults without mental disorders have only emerged in recent years [34]. Thus, the aim of the current systematic review and meta-analysis was to investigate the effects of MBI involving meditative movements on psychological health outcomes among older adults without mental health conditions in order to evaluate a potential reduction of psychological symptoms promoting the prevention of late-life mental disorders. To the best of the authors’ knowledge, no previous systematic review with meta-analysis, including only randomized controlled trials, has addressed the effects of MBI involving meditative movements on various psychological health outcomes (i.e., quality of life, depressive symptoms, fear of falling and sleep quality) in older people without mental illnesses.

Since quality of life is a very common parameter assessed secondarily or tertiarily, previous studies reported comparable results [18]. However, an interesting trend could be observed concerning the subscale ‘psychological functioning’. Tai Chi/Qigong (TCQ) seem to be more effective for improving psychological aspects of quality of life than Yoga/Pilates (YP). When comparing the included studies in terms of physical effort, the performed exercises of YP were more physically demanding as strengthening and balance exercises were often part of the training programs. Nevertheless, both groups showed comparable results in the self-reported subscale ‘physical functioning’ leading to the assumption that YP may improve physical fitness more than TCQ, but the subjective assessment of physical functioning may be enhanced by YP as well as TCQ. Comparing all three derived subdimensions of quality of life, larger effect sizes can be seen in psychological and physical functioning. This implies that the positive effect of MBI involving meditative movements on quality of life may not be attributable to social stimuli. Still, even though all interventions were group based, only participants of TCQ seem to benefit regarding socialization. However, this result should not be overestimated and generalized because of two facts. On the one hand, only four studies comprised YP and on the other hand, control groups of two studies [48,73] focused on socialization, which led to an overall shift in the direction of the control group. Social support still has not yet been investigated solely, but positive effects are estimated [82]. In conclusion, it should be kept in mind that the heterogeneity of the influencing factors of the included studies (e.g., sample size) were again increased by various factors of the multidimensional construct ‘quality of life’.

Given that depression is one of the most prevalent mental illnesses among older adults, studies focusing on improvements of depressive symptoms are becoming increasingly common [21]. The present meta-analysis gathered a significantly positive overall effect for depressive symptoms (*p* = 0.007) that underlines the previous state of research in younger populations [6,17]. YP seem to be more beneficial for reducing depressive symptoms, but both subgroups showed a large heterogeneity in effect sizes that limits generalizability. Nonetheless, effect sizes are in the same range with the ones reported in the past among the adult population [21]. The larger effect size for Tai Chi found in a previous study focusing on Chinese older people [22] compared to that found in our study may suggest that Tai Chi is more efficacious in Chinese older adults in contrast to their counterparts from other ethnicities. Since TCQ are rooted in Chinese culture, TCQ may be more accepted by older Chinese adults. This could be one reason of the low attendance rates in some included studies [47,76,81]. For example, Fransen et al. [47] reported a higher class attendance for hydrotherapy with 81% attending at least half of the available 24 classes, compared with just 61% for Tai Chi.

Investigators have found that Tai Chi has been associated with a decrease in number of falls, the risk for falling and in fear of falling, particularly in frailer, higher fall-risk older adults [83]. It was striking that in the two studies that comprised older adults at risk of falling [60,75] an expected higher effect size for fear of falling could not be observed, but effect sizes of *g* = 0.24 and *g* = 0.41 situated in the middle range. On the other hand, two studies [62,63] influenced the overall effect in a positive way having extremely large effect sizes compared to the others (*g* = 1.93; *g* = 2.30). However, the causality could not be identified when looking more precisely at these studies. Even though fear of falling has a psychological origin, both physical fitness and mental health can be affected by MBI involving meditative movements and must even be affected for favorable long-term handling.

There is positive evidence to suggest benefit of MBI involving meditative movements for sleep quality as in previous studies [29,31]. Wu et al. [29] even reported a moderate effect on improving sleep quality that may be due to the investigated population. The included sample was limited to older adults with poor sleep quality compared to the present work that made no restrictions regarding sleep quality. One included study [56] involved older adults with moderate sleep complaints. Surprisingly, this was the only study with an effect size favoring control group (*g* = −0.25). However, when looking more carefully at the study sample, it will be noted that older adults with moderate sleep complaints (defined as Pittsburgh Sleep Quality Index (PSQI) ≥ 5) as well as without sleep complaints (PSQI < 5) were included. Among the participants who entered the study with poor sleep quality, significant improvements were found in the experimental group relative to the control group (health education). The group performing Tai Chi Chih (PSQI ≥ 5) even continued to improve after the intervention (9-week follow-up), whereas the control group worsened. No positive effects were found among older adults with good sleep quality, which could explain the overall shift in favor of the control group. Nevertheless, the interpretation of the actual findings is limited due to the number of included studies, above all for YP. For this reason, the impacts of MBI involving meditative movements on sleep quality in older people without mental disorders need to be a purpose of future high-quality randomized controlled trials.

The significant subgroup difference of exercising 1–2 times per week compared to three times or more in the outcomes ‘quality of life’ and ‘depressive symptoms’ (*p* = 0.03; *p* = 0.004) revealed a dependency of training frequency. Similar findings of the meta-analysis of Nebiker et al. [84] underpin the increasing effect with growing exercise frequency on depressive symptoms. Fear of falling, in contrast, seems to be rather independent of training frequency (*p* = 0.88). The analyses of all quality of life subdimensions and sleep quality for YP are not very representative because the number of studies was too small and thus not further interpreted.

### 4.1. Strengths and Limitations

The present meta-analytical review was performed in accordance with the PRISMA guidelines [38]. An essential strength of the performed analyses is the provision of a summary of the interaction between various psychological health outcomes and several mind–body interventions (MBI) involving meditative movements among older adults aged 59 years or older. To ensure a high methodological quality, study selection was restricted to randomized controlled trials and a PEDro score of at least five, which can be seen as a key strength. Regardless of these advantages, some limitations must be considered. First, the effect sizes could have been affected by lack of concealment (22 out of 37 studies), inability to blind therapists/subjects (all studies) or lack of blinding assessors (16 out of 37 studies). Second, the search strategy in the present study was limited to English language publications. Knowing that many studies of MBI involving meditative movements that often have their origin in Asia are published in a foreign language only, a potential language bias cannot be neglected. Third, no librarian was consulted to design the search strategy, which resulted in a potentially limited search strategy. In addition, a heterogeneity in study characteristics was present. The analyzed population differed in terms of setting (community dwelling vs. not community dwelling) and physical health status. The sample size ranging from *n* = 17 to *n* = 269, the intervention length (range from 4 weeks to 52 weeks) of the included studies as well as the frequency (range from 1× to 5×/week) showed a broad spectrum, too. Perhaps the inclusion criteria should have defined a required intervention duration and a minimal sample size. Furthermore, inactive control groups (e.g., wait list, health education or usual care) were included, which can be seen as another limitation. Treatment usually involves some potentially effective components that may be absent in no-treatment controls (e.g., positive encouragement) [85]. This point should be considered when interpreting findings. Future systematic reviews or meta-analyses should include more unpublished trials and quasi-experimental design studies to minimize publication bias and the selection bias of systematic reviews. Moreover, in the majority of the studies the description of the intervention contents was insufficient (e.g., intensity not mentioned), so that the understanding and comparison are limited. The exercises within the same intervention varied as well, which complicates generalizability and recommendations in terms of exercise modalities. Besides, the small number of studies for several outcomes, above all in the subgroup Yoga/Pilates, and the small effect sizes (apart from fear of falling) warrant caution in the interpretation of the results. Another limitation is the missing subgroup analysis for trials comprising orthopedic conditions/concerns (6 out of 37 studies). Hence, this could be a subject for future research.

### 4.2. Implications and Considerations

A large heterogeneity in parameters of mental health is predominant in the examined field of study. There are huge differences in the amount of measuring instruments for each outcome, as well as in their number of items and/or subscales. Since mental health issues are prevalent in older adults, it is necessary to utilize future resources on the development of a consistent assessment to simplify data collection and capture a holistic evaluation of psychological health among older people. Quality of life is one of the most frequently used outcomes of mental health in prior studies of MBI, but allocated subscales, their coherences and the underlying conceptualization differ and are rather unclear. The dimension ‘quality of life’ is therefore supposed to be split up to evaluate the effects on its subscales and their interrelations leading to a detailed overall picture. As a result, interventions can be tailored to the specific needs and demands of older people.

The present heterogeneity of the included populations and exercise modalities does not allow making concise recommendations. Nevertheless, Tai Chi, Qigong, Yoga and Pilates seem to positively influence quality of life, depressive symptoms, fear of falling and sleep quality in older adults without mental health conditions, regardless of intervention type. Tai Chi and Qigong are applied more frequently in practice, which results in a stronger evidence base at present. But Yoga and Pilates are arousing growing interest regarding late-life exercise. The ability of MBI involving meditative movements to foster states of relaxation and decrease body tension is clearly in line with behaviors linked to improved psychological health [6,31]. The underlying efficacy in psychological health outcomes may be based on their effects on stress reduction and the provision of self-care and self-management strategies [31]. Most MBI involving meditative movements can even be practiced at home (low risk), making them accessible and attractive to people with a wide range of ages, comorbidities and socioeconomic backgrounds.

Future research should still concentrate on the impact of MBI involving meditative movements in older people without versus with mental health conditions to draw comparisons across non-clinical and clinical populations. In the latter case, perhaps even larger effects may be observed in terms of psychological health outcomes, since a predominant tendency towards improvement of the examined outcomes was demonstrated in older adults without mental disorders by the present work. This implies the necessity of perceiving MBI involving meditative movements as a future research topic to further confirm these types of exercise as a potential cost-effective, non-pharmacological, preventive method for counteracting the increase of mental disorders among older adults [4]. These interventions may offer an age-appropriate possibility of improving psychological health through various levels and simultaneously incorporating more movement into daily life. Low demands and the variability of exercises make them a promising approach among older people that needs to be exploited to its fullest extent in the future.

## 5. Conclusions

Mind–body interventions (MBI) involving meditative movements showed promising signs of improvements in psychological health (i.e., quality of life, depressive symptoms, fear of falling and sleep quality) among older adults without mental disorders. Even though Tai Chi and Qigong (TCQ) are more often applied than Yoga and Pilates (YP), significant positive effects on various psychological health outcomes could be observed in both subgroups. Furthermore, the effect sizes of quality of life and depressive symptoms seem to be dependent on training frequency, whereas those of fear of falling seem independent.

The predominant heterogeneity in study characteristics, especially in the selected population, and other limitations warrant caution in the interpretation of the current results. However, MBI involving meditative movements—above all, Yoga and Pilates—need greater emphasis in future gerontological research and should be further confirmed as possible age-appropriate preventive measures in order to counteract the increase of late-life mental disorders. In this way, the evidence base of these interventions affecting not simply two levels—cognitive and physical functioning—but also various domains of psychological functioning among older adults may be strengthened.

## Figures and Tables

**Table 1:** Search levels and terms of the literature search.

Search Levels	Search Terms with Boolean Operators
Search #1	((psychological AND (well-being OR health)) OR well-being OR wellness OR quality of life OR mindful* OR self-efficacy OR self-esteem OR fear of falling OR balance confidence OR depression OR depressive symptoms OR sleep quality OR ((emotional OR mental) AND health))
Search #2	AND (mind body OR tai chi OR qigong OR yoga OR pilates OR feldenkrais)
Search #3	AND (old* OR elder* OR senior* OR aged)

Note. The asterisk (*) represents any group of characters, including no character.

**Table 2:** Classification of subscales of the SF-36/12, LEIPAD, MFI, NHP, WHOQOL-BREF.

	Subscales of Questionnaires		
Questionnaires	Physical Functioning	Psychological Functioning	Social Functioning
SF-36/12	Physical functioning Role—physical Body pain General health	Mental health Role—emotional Vitality	Social functioning
LEIPAD	Physical functioning Self-care	Mental functioning	Social functioning
MFI	Physical fatigue Reduced activity	Mental fatigue Reduced motivation	---
NHP	Physical abilities	Tiredness Emotional reaction	Social isolation
WHOQOL-BREF	Physical health	Psychological health	Social relations

Notes. SF-36: 36-Item Short Form Health Survey; SF-12: 12-Item Short Form Health Survey; LEIPAD: Leiden-Padua questionnaire; MFI: Multidimensional Fatigue Inventory; NHP: Nottingham Health Profile; WHOQOL-BREF: World Health Organization Quality of Life Brief Version.

## Supplementary Materials

The following are available online at https://www.mdpi.com/1660-4601/17/18/6556/s1, Figure S1: Funnel plots for publication bias assessment on the outcome quality of life (A) and its sub-scales: physical functioning (B), psychological functioning (C) and social functioning (D), Figure S2: Funnel plots for publication bias assessment on the outcomes depressive symptoms (E), fear of falling (F) and sleep quality (G), Table S1: Characteristics of the included studies, Table S2: Questionnaires and corresponding outcomes of the included studies, Table S3: PEDro scores and sum of the included trials. References [86,87,88,89,90,91,92,93,94,95,96,97,98,99,100,101,102,103,104,105,106,107,108,109,110,111,112,113,114,115] are cited in the Supplementary Materials.
