# Supplementary material for: Effects of Mind–Body Interventions Involving Meditative Movements on Quality of Life, Depressive Symptoms, Fear of Falling and Sleep Quality in Older Adults: A Systematic Review with Meta-Analysis
Source: Int J Environ Res Public Health. 2020 Sep 9;17(18):6556. doi: 10.3390/ijerph17186556 (PMC7559727; doi:10.3390/ijerph17186556)
Supplement: Supplementary file 1 [file ijerph-17-06556-s001.pdf]

[IJERPH]

Supplementary Materials

Effects of Mind–Body Interventions Involving Meditative Movements on Quality of Life, Depressive Symptoms, Fear of Falling and Sleep Quality in Older Adults: A Systematic Review with Meta-Analysis

M. Weber, T. Schnorr, M. Morat, T. Morat, L. Donath

Included elements in this document: 2 Figures and 3 Tables

Number	Page(s)	Title
Figure S1	1	Funnel plots for publication bias assessment on the outcome quality of life (A) and its subscales: physical functioning (B), psychological functioning (C) and social functioning (D). The middle-dashed line indicated the mean SMD. SE: standard error; SMD: standardized mean difference.
Figure S2	1	Funnel plots for publication bias assessment on the outcomes depressive symptoms (E), fear of falling (F) and sleep quality (G). The middle-dashed line indicated the mean SMD. SE: standard error; SMD: standardized mean difference.
Table S1	2–11	Characteristics of the included studies.
Table S2	12–17	Questionnaires and corresponding outcomes of the included studies.
Table S3	18–20	PEDro scores and sum of the included trials.

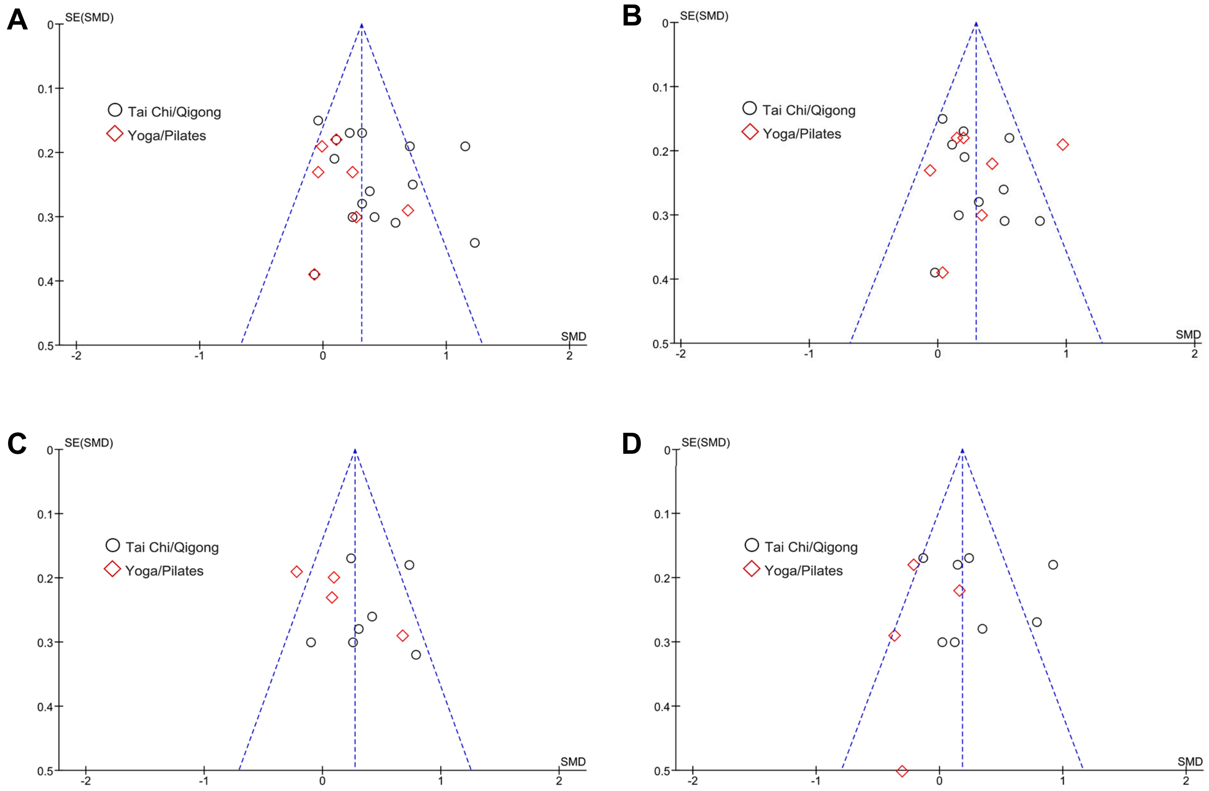

Figure S1 Funnel plots for publication bias assessment on the outcome quality of life (A) and its subscales: physical functioning (B), psychological functioning (C) and social functioning (D). The middle-dashed line indicated the mean SMD. SE: standard error; SMD: standardized mean difference.

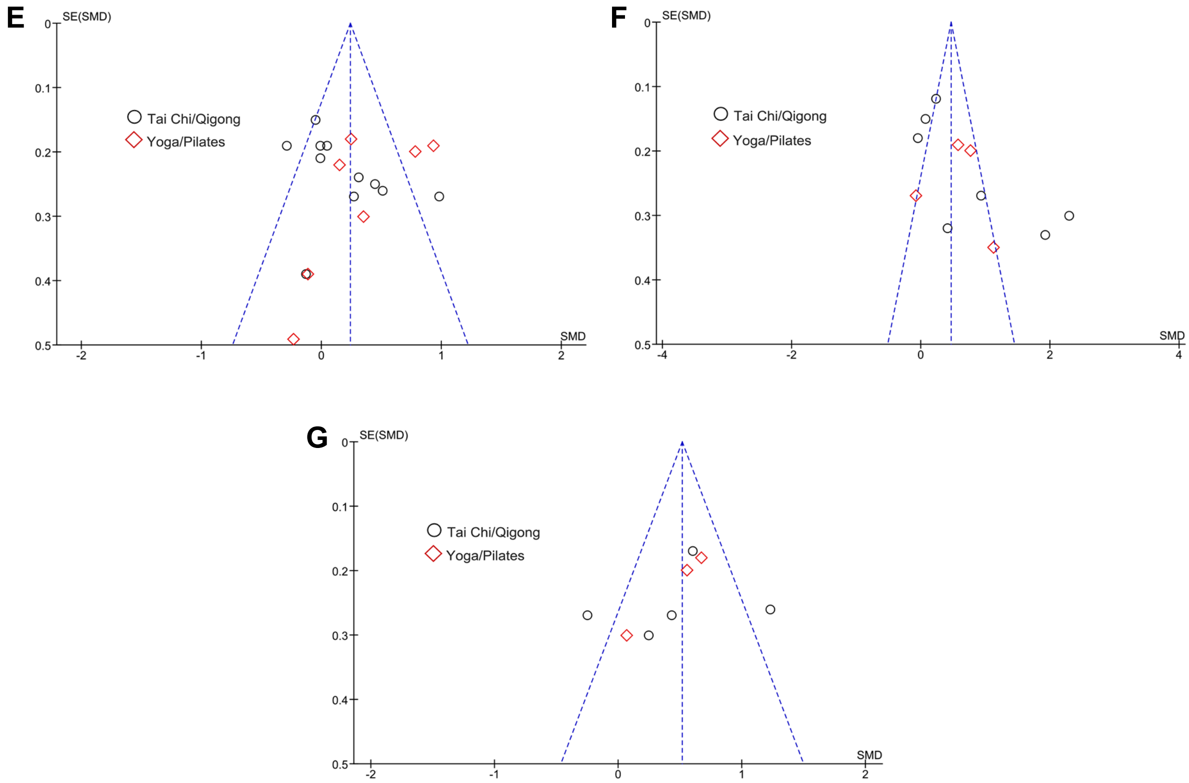

Figure S2 Funnel plots for publication bias assessment on the outcomes depressive symptoms (E), fear of falling (F) and sleep quality (G). The middle-dashed line indicated the mean SMD. SE: standard error; SMD: standardized mean difference.

Table S1 Characteristics of the included studies.

Reference: Publication Year, Country	Study Design	Sample: Population, Sample Size, Mean Age ± *SD*	Groups: Sample Size, Mean Age ± *SD*	Intervention	Training Characteristics	Target Outcome Measures	PEDro Score
[1] 2019, Spain	Two-arm RCT	Community-dwelling postmenopausal women aged 60 and above, *n* = 107, 68.2 ± 8.4 years	IG *n* = 55, 70.0 ± 7.8 years	(a) IG: Pilates (basic principles, breathing, strengthening and stretching exercises for main body segments)	12 weeks, 2 sessions/week, 60 min/session	HADS PSQI	7
			CG *n* = 52, 66.8 ± 10.1 years	(b) CG: day-to-day lifestyle, received a series of guidelines			
[2] 2019, Spain	Two-arm RCT	Community-dwelling postmenopausal women aged 60 and above, *n* = 107, 68.2 ± 8.4 years	IG *n* = 55, 70.0 ± 7.8 years	(a) IG: Pilates (basic principles, breathing, strengthening and stretching exercises for main body segments)	12 weeks, 2 sessions/week, 60 min/session	ABC FES-I	8
			CG *n* = 52, 66.8 ± 10.1 years	(b) CG: day-to-day lifestyle, received a series of guidelines			
[3] 2009, Turkey	Two-arm RCT	Sedentary women with senile osteoporosis aged 65 and above, *n* = 44, 70.2 ± 5.6 years	IG *n* = 22, 69.5 ± 4.9 years	(a) IG: Tai Chi	6 months, 3 sessions/week, weeks 1-4: 30min/session weeks 5-24: 60 min/session	NHP SF-36	7
			CG *n* = 22, 71.2 ± 6.3 years	(b) CG: normal lifestyle			

Table S1 *Continued*

Reference: Publication Year, Country	Study Design	Sample: Population, Sample Size, Mean Age ± *SD*	Groups: Sample Size, Mean Age ± *SD*	Intervention	Training Characteristics	Target Outcome Measures	PEDro Score
[4] 2013, China	Three-arm RCT	Chronic obstructive pulmonary disease (COPD) patients aged 60 and above, *n* = 206	IG *n* = 70, 71.7 ± 8.2 years	(a) IG: Tai Chi Qigong	3 months, 2 sessions/week, 60 min/session	SGRQ MSPSS	6
			CG *n* = 67, 73.6 ± 7.4 years	(b) CG: usual care			
			BW *n* = 69, 73.6 ± 7.5 years	(c) BW: breathing and walking exercise, not included in meta-analysis			
[5] 2017, China	Two-arm RCT	"Hidden" (isolated) elderly aged 60 and above, *n* = 46, 77.3 ± 7.4 years	IG *n* = 24, 75.4 ± 5.9 years	(a) IG: Tai Chi Qigong (18 forms)	3 months, 2 sessions/week, 60 min/session	SF-12 MHI-18 LSNS-6 DJGLS RSSQ RSES	6
			CG *n* = 22, 79.4 ± 8.5 years	(b) CG: routine activities			
[6] 2009, China	Two-arm cluster RCT	Community-dwelling older adults aged 60 and above, *n* = 128, 69.2 ± 6.2 years	IG *n* = 62, 65.8 ± 4.3 years	(a) IG: silver Yoga	6 months, 3 sessions/week, 70 min/session	SF-12 TDQ PSQI	5
			CG *n* = 66, 72.4 ± 6.0 years	(b) CG: wait-list control group			
[7] 2010, USA	Two-arm RCT	Postmenopausal women with osteopenia aged 65 and above, *n* = 53	IG *n* = 26, 72.4 ± 6.2 years	(a) IG: Tai Chi (simplified 24-form Yang-style)	24 weeks, 3 sessions/week, 60 min/session	SF-36	6
			CG *n* = 27, 71.3 ± 6.0 years	(b) CG: no treatment			

Table S1 *Continued*

Reference: Publication Year, Country	Study Design	Sample: Population, Sample Size, Mean Age ± *SD*	Groups: Sample Size, Mean Age ± *SD*	Intervention	Training Characteristics	Target Outcome Measures	PEDro Score
[8]<br>2013,<br>Korea	Three-arm RCT	Single women with backache aged 65 and above,<br>*n* = 102,<br>68.5 years	YJJ<br>*n* = 32	(a) YJJ: Yi Jin Jing (health Qigong), 12 routines	12 weeks,<br>3 sessions/week,<br>60 min/session	GDS-SF	5
			LZJ<br>*n* = 36	(b) LZJ: Liu Zi Jue (health Qigong), 6 routines			
			CG<br>*n* = 34	(c) CG: no treatment			
[9]<br>2017,<br>Iran	Two-arm RCT	Residents of elderly home aged 60 and above,<br>*n* = 56	IG<br>*n* = 27,<br>69.2 ± 5.5 years	(a) IG: Tai Chi (10 steps)	12 weeks,<br>3 sessions/week,<br>5-25 min/session (progressive duration)	BDI-II	5
			CG<br>*n* = 29,<br>69.3 ± 5.0 years	(b) CG: activities of daily living (ADL)			
[10]<br>2020,<br>China + Canada	Two-arm cluster RCT	Adults with sleep disturbances aged 60 and above,<br>*n* = 139,<br>71.1 ± 6.3 years	IG<br>*n* = 67,<br>70.3 ± 5.7 years	(a) IG: Baduanjin (Qigong)	24 weeks,<br>5 sessions/week,<br>45 min/session	SF-36<br>PSQI	6
			CG<br>*n* = 72,<br>71.8 ± 6.7 years	(b) CG: wait-list control group			
[11]<br>2007,<br>Australia	Three-arm RCT	Community-dwelling adults with symptomatic hip or knee osteoarthritis aged between 59 and 85,<br>*n* = 152,<br>71.1 ± 6.3 years	IG<br>*n* = 56,<br>70.8 ± 6.3 years	(a) IG: Tai Chi for arthritis (modification of 24 forms from the Sun style of Tai Chi)	12 weeks,<br>2 sessions/week,<br>60 min/session	SF-12<br>DASS21	8
			CG<br>*n* = 41,<br>69.6 ± 6.1 years	(b) CG: wait-list control group			
			HT<br>*n* = 55,<br>70.0 ± 6.3 years	(c) HT: hydrotherapy, not included in meta-analysis			

Table S1 *Continued*

Reference: Publication Year, Country	Study Design	Sample: Population, Sample Size, Mean Age ± *SD*	Groups: Sample Size, Mean Age ± *SD*	Intervention	Training Characteristics	Target Outcome Measures	PEDro Score
[12] 2016, Israel	Two-arm RCT	Community-dwelling older adults aged 65 and above, *n* = 78, 71.15 ± 4.3 years	IG *n* = 34, 70.3 ± 3.8 years	(a) IG: Pilates (balance control, postural control, strength, 3 levels; Thera-Band + Swiss balls)	3 months, 3 sessions/week, 60 min/session	SF-36	7
			CG *n* = 44, 72.1 ± 4.6 years	(b) CG: no treatment			
[13] 2009, USA	Two-arm RCT	Older adults aged 60 and above with kyphosis angle ≥ 40° (noticed after age 50), *n* = 118, 75.5 ± 7.4 years	IG *n* = 58, 74.5 ± 7.6 years	(a) IG: Hatha Yoga [*asanas* (poses) and *pranas* (breathing)]	24 weeks, 3 sessions/week, 60 min/session	SF-36 ABC	8
			CG *n* = 60, 76.5 ± 7.2 years	(b) CG: monthly lunch/seminars (2 hours per session)			
[14] 2018, USA	Two-arm RCT	Inactive adults at risk for further mobility disability aged between 60 and 89, *n* = 45	IG *n* = 22, 71.6 ± 8.3 years	(a) IG: silver age Yoga (program based on principles of Iyengar Yoga)	10 weeks, 2 sessions/week, 60 min/session	SF-36 CES-D PSQI	6
			HE *n* = 23, 76.0 ± 7.8 years	(b) HE: health education (weekly information workshops)			
[15] 2011, Iran	Two-arm RCT	Residents of elderly home aged 60 and above, *n* = 56	IG *n* = 29, 68.7 ± 5.5 years	(a) IG: Tai Chi (10 stages)	12 weeks, 3 sessions/week, 5-25 min/session (progressive duration)	PSQI	5
			CG *n* = 27, 69.4 ± 5.3 years	(b) CG: activities of daily living (ADL)			

Table S1 *Continued*

Reference: Publication Year, Country	Study Design	Sample: Population, Sample Size, Mean Age ± *SD*	Groups: Sample Size, Mean Age ± *SD*	Intervention	Training Characteristics	Target Outcome Measures	PEDro Score
[16] 2018, Iran	Two-arm RCT	Community-dwelling adults aged 60 and above, *n* = 60, estimated mean age 71.5 years	IG *n* = 30	(a) IG: Tai Chi Chuan (Yang-style)	8 weeks, 2 sessions/week, 55 min/session	FES-I	7
			CG *n* = 30	(b) CG: no treatment			
[17] 2016, China + Australia	Two-arm RCT	Adults in a long-term care facility using wheelchairs aged 65 and above, *n* = 60, 81.3 ± 8.1 years	IG *n* = 30, 80.7 ± 9.7 years	(a) IG: STEP; simplified Tai Chi exercise program (seated Tai Chi)	26 weeks, 3 sessions/week, 40 min/session	POMS-SF	6
			CG *n* = 30, 81.8 ± 6.3 years	(b) CG: usual care activities			
[18] 2016, China + Australia	Two-arm RCT	Adults in a long-term care facility using wheelchairs aged 65 and above, *n* = 60, 81.3 ± 8.1 years	IG *n* = 30, 80.7 ± 9.7 years	(a) IG: STEP; simplified Tai Chi exercise program (seated Tai Chi)	26 weeks, 3 sessions/week, 40 min/session	WHOQOL-BREF GDS-SF	6
			CG *n* = 30, 81.8 ± 6.3 years	(b) CG: usual care activities			
[19] 2011, China	Three-arm RCT	Community-dwelling adults aged 60 and above, *n* = 186, estimated mean age 68.5 years	IG *n* = 62	(a) IG: Tai Chi + CB (core of lessons: 10 positions derived from the Yang-style and 8-weekly sessions of cognitive-behavioral strategies)	8 weeks, 3-5 sessions/week, 60 min/session	WHOQOL-BREF ISSB GFFM FES	8
			CG *n* = 62	(b) CG: no treatment			
			CB *n* = 62	(c) CB: cognitive-behavioral strategies, not included in meta-analysis			

Table S1 *Continued*

Reference: Publication Year, Country	Study Design	Sample: Population, Sample Size, Mean Age ± *SD*	Groups: Sample Size, Mean Age ± *SD*	Intervention	Training Characteristics	Target Outcome Measures	PEDro Score
[20] 2008, USA	Two-arm RCT	Adults with moderate sleep complaints aged 59 and above, *n* = 112	IG *n* = 59, 69.7 ± 6.2 years	(a) IG: Tai Chi Chih (specific set of 20 exercises)	16 weeks, 3 sessions/week, 40 min/session	BDI PSQI	7
			HE *n* = 53, 70.2 ± 7.5 years	(b) HE: health education (16 didactic presentations)			
[21] 2001, USA	Two-arm RCT	Healthy, physically inactive adults aged 65 and above, *n* = 72, 72.8 ± 5.1 years	IG *n* = 40, 72.8 ± 4.7 years	(a) IG: Tai Chi (classical Yang-style; 24 forms; strength, balance, postural alignment and concentration)	6 months, 2 sessions/week, 60 min/session	SF-20	5
			CG *n* = 32, 72.7 ± 5.7 years	(b) CG: wait-list control group			
[22] 2019, USA	Two-arm cluster RCT	Adults from subsidized housing facilities aged 60 and above, *n* = 180, 75.3 ± 8.8 years	IG *n* = 93, 75.9 ± 9.1 years	(a) IG: Tai Chi (9 core movements following the traditional Cheng-Man-Ch'ing's Yang-style)	52 weeks, 2 sessions/week, 60 min/session	SF-12 CES-D ABC	6
			HE *n* = 87, 74.6 ± 8.6 years	(b) HE: health education (lecture and group discussions)			
[23] 2009, The Netherlands	Two-arm RCT	Community-dwelling adults with high fall risk aged 70 and above, *n* = 269, 77 ± 4.7 years	IG *n* = 138, 77.5 ± 4.7 years	(a) IG: Tai Chi Chuan (10 positions from Yang-style in main exercise + warm-up from Chi Kung)	13 weeks, 2 sessions/week, 60 min/session	FES	8
			CG *n* = 131, 76.8 ± 4.6 years	(b) CG: usual care			

Table S1 *Continued*

Reference: Publication Year, Country	Study Design	Sample: Population, Sample Size, Mean Age ± *SD*	Groups: Sample Size, Mean Age ± *SD*	Intervention	Training Characteristics	Target Outcome Measures	PEDro Score
[24] 2017, China	Two-arm RCT	Women with knee osteoarthritis aged 60 and above, *n* = 46, 71.1 ± 2.7 years	IG *n* = 23, 64.6 ± 3.4 years	(a) IG: Tai Ji Quan (8 forms adapted primarily from the 24-form practice routine)	24 weeks, 3 sessions/week, 60 min/session	SF-36 PSQI	7
			HE *n* = 23, 64.5 ± 3.4 years	(b) HE: bi-weekly health education classes			
[25] 2018, Iran	Two-arm RCT	Community-dwelling adults aged 60 and above, *n* = 53	IG *n* = 27, 67.2 ± 5.4 years	(a) IG: Tai Chi (Yang-style)	10 weeks, 3 sessions/week, 20 min/session	FES-I	6
			CG *n* = 26, 68.1 ± 5.2 years	(b) CG: normal life routine			
[26] 2012, Vietnam + Germany	Two-arm RCT	Community-dwelling adults aged 60 and above, *n* = 73, 69.0 ± 5.1 years	IG *n* = 39, 69.2 ± 5.3 years	(a) IG: Tai Chi (24-form exercise; balance, postural alignment, concentration)	6 months, 2 sessions/week, 60 min/session	PSQI FES	5
			CG *n* = 34, 68.7 ± 5.0 years	(b) CG: routine of daily activities			
[27] 2016, Iran	Two-arm RCT	Community-dwelling adults aged between 60 and 74, *n* = 39	IG *n* = 20, 68.0 ± 4.9 years	(a) IG: Hatha Yoga (emphasis on *Pavanamuktasana* and balance movements)	8 weeks, 2 sessions/week, 60 min/session	MFES	5
			CG *n* = 19, 68.8 ± 4.8 years	(b) CG: no treatment			

Table S1 *Continued*

**Reference: Publication Year, Country**	**Study Design**	**Sample: Population, Sample Size, Mean Age ± *SD***	**Groups: Sample Size, Mean Age ± *SD***	**Intervention**	**Training Characteristics**	**Target Outcome Measures**	**PEDro Score**
[28] 2017, Australia + Thailand	Three-arm RCT	Healthy, physically inactive adults aged 60 and above, *n* = 39, 67.7 ± 6.7 years	TY *n* = 13, 67.7 ± 4.9 years	(a) TY: Thai Yoga (15 postures)	12 weeks, 2 sessions/week, 80 min/session	SF-36 CES-D	8
			TC *n* = 13, 67.2 ± 8.3 years	(b) TC: Tai Chi (Sun Style; 12 move-ments)			
			CG *n* = 13, 65.2 ± 6.7 years	(c) CG: telephone calls (information on exercise)			
[29] 2019, Korea	Two-arm RCT	Community-dwelling adults aged 65 and above, *n* = 42	IG *n* = 20, 71.6 ± 6.0 years	(a) IG: Tai Chi (24 forms of Yang-style)	6 weeks, 5 sessions/week, 60 min/session	SWLS PEBS	6
			CG *n* = 22, 70.6 ± 7.0 years	(b) CG: no treatment			
[30] 2006, USA	Three-arm RCT	Inactive, community-dwelling adults aged 65 and above, *n* = 118	IG *n* = 38, 71.5 ± 4.9 years	(a) IG: Iyengar Yoga	6 weeks, 1 session/week, 90 min/session	SF-36 MFI CESD-10 POMS	6
			CG *n* = 42, 73.6 ± 5.1 years	(b) CG: wait-list control group			
			AE *n* = 38, 71.2 ± 4.4 years	(c) AE: aerobic exercise, not included in meta-analysis			

Table S1 *Continued*

Reference: Publication Year, Country	Study Design	Sample: Population, Sample Size, Mean Age ± *SD*	Groups: Sample Size, Mean Age ± *SD*	Intervention	Training Characteristics	Target Outcome Measures	PEDro Score
[31] 2018, UK + Iran + Norway	Two-arm RCT	Healthy, inactive men aged 60 and above living in a retirement home, *n* = 132, 67.5 ± 0.3 years	IG *n* = 66, 67.9 ± 4.4 years	(a) IG: Tai Chi (Yang-style; 10 movements extracted from 24 simple forms of Tai Chi Chuan)	8 weeks, 3 sessions/week, 30-40 min/session (progressive duration)	LEIPAD	8
			CG *n* = 66, 67.0 ± 3.6 years	(b) CG: no treatment			
[32] 2016, Germany + Switzerland	Three-arm RCT	Adults with chronic low back pain aged 65 and above (community-dwelling and retirement homes), *n* = 176	YG *n* = 61, 73.0 ± 5.6 years	(a) YG: Viniyoga method; physical, breathing and concentration exercises while sitting, standing and lying	3 months, YG: 2 sessions/week, 45 min/session, QG: 1 session/week, 90 min/session	SF-36 GDS	7
			QG *n* = 58, 72.4 ± 5.7 years	(b) QG: Dantian and Nei Yang Gong (Qigong) exercises from the Training System Liu Ya Fei			
			CG *n* = 57, 72.6 ± 6.0 years	(c) CG: wait-list control group			
[33] 2017, UK	Two-arm RCT	Physically inactive adults aged 60 and above, *n* = 52, 74.8 ± 7.2 years	IG *n* = 25, 73.8 ± 6.5 years	(a) IG: Yoga (*asana* and *pranayama*; postural advice, breath work, concentration)	10 weeks, 1 session/week, 75 min/session	EQ-5D-5L WEMWBS	7
			CG *n* = 27, 75.7 ± 7.9 years	(b) CG: wait-list control group			
[34] 2013, Australia	Two-arm RCT	Community-dwelling adults aged 59 and above, *n* = 54, 68.0 ± 7.1 years	IG *n* = 27, 67.7±7.2 years	(a) IG: Iyengar Yoga	12 weeks, 2 sessions/week, 60 min/session	Short FES-I	8
			CG *n* = 27, 67.3 ± 6.1 years	(b) CG: no treatment			

Table S1 *Continued*

Reference: Publication Year, Country	Study Design	Sample: Population, Sample Size, Mean Age ± *SD*	Groups: Sample Size, Mean Age ± *SD*	Intervention	Training Characteristics	Target Outcome Measures	PEDro Score
[35] 2013, China	Two-arm RCT	Frail adults aged 60 and above, *n* = 116	IG *n* = 61, 83.3 ± 6.3 years	(a) IG: Qigong (Yan Chai Yi Ji; ten-section brocade for standing and sitting positions)	12 weeks, 2 sessions/week, 60 min/session	GDS	5
			CG *n* = 55, 84.9 ± 6.0 years	(b) CG: newspaper reading group			
[36] 2010, USA	Two-arm RCT	Community-dwelling adults aged 60 and above, *n* = 17	IG *n* = 7, 75.5 ± 9.2 years	(a) IG: Yoga	4 weeks, 2 sessions/week, 60 min/session	UCLA CES-D	5
			CG *n* = 10, 74.5 ± 8.1 years	(b) CG: socialization group			
[37] 2016, China	Three-arm RCT	Community-dwelling non-fallers at risk of falling aged between 65 and 74, *n* = 61	TC *n* = 20, 68.8 ± 3.0 years	(a) TC: Tai Chi (Yang-style)	16 weeks, 3 sessions/week, 90 min/session	FES-I	8
			CG *n* = 21, 69.9 ± 3.3 years	(b) CG: no treatment			
			ExBP *n* = 20, 70.2 ± 3.9 years	(c) ExBP: Exercise for Balance improvement program, not included in meta-analysis			

Notes. n: sample size; SD: standard deviation; IG: intervention group; CG: control group; PEDro: Physiotherapy Evidence Database scale; ABC: Activities-Specific Balance Confidence Scale; BDI: Beck Depression Inventory; BDI-II: Beck Depression Inventory-II; CES-D: Center for Epidemiological Studies Depression Scale; DASS21: Depression Anxiety Stress Scale-21; DJGLS: De Jong Gierveld Loneliness Scale; EQ-5D-5L; EuroQoL 5-dimension 5-level; (Short) FES: (Short) Falls Efficacy Scale; FES-I: Falls Efficacy Scale-International; GDS: Geriatric Depression Scale; GDS-SF: Geriatric Depression Scale-Short Form; GFFM: Geriatric Fear of Falling Measure; HADS: Hospital Anxiety and Depression Scale; ISSB: Inventory of Social Supportive Behaviors; LEIPAD: Leiden-Padua questionnaire; LSNS-6: Lubben Social Network Scale-6; MFES: Modified Falls Efficacy Scale; MFI: Multidimensional Fatigue Inventory; MHI-18: Mental Health Inventory-18; MSPSS: Multidimensional Scale of Perceived Social Support; NHP: Nottingham Health Profile; PEBS: Perceived Self-Efficacy Scale; POMS: Profile of Mood States; POMS-SF: Profile of Mood States-Short Form; PSQI: Pittsburgh Sleep Quality Index; RSES: Rosenberg Self-Esteem Scale; RSSQ: Revised Social Support Questionnaire; SF-36: 36-Item Short Form Health Survey; SF-12: 12-Item Short Form Health Survey; SF-20: 20-Item Short Form Health Survey; SGQR: St. George's Respiratory Questionnaire; SWLS: Satisfaction with Life Scale; TDQ: Taiwanese Depression Questionnaire; UCLA: University of California, Los Angeles, Loneliness Scale; WEMWBS: Warwick-Edinburgh Mental Well-being Scale; WHOQOL-BREF: World Health Organization Quality of Life Brief Version.

Table S2 Questionnaires and corresponding outcomes of the included studies.

Name<br>(Abbreviation, Reference)	Items	Dimension/Subscales	Interpretation
		**Overall Quality of life (QoL)**	
EuroQol 5-dimension 5-level<br>(EQ-5D-5L)<br>[38]	5 + VAS	QoL<br>*mobility*<br>*self-care*<br>*usual activities*<br>*pain/discomfort*<br>*anxiety/depression*<br>*visual analogue scale (VAS)*	Total score: 0–1 (index value)<br>Total score: 0–100 (VAS)<br>Higher scores indicate better QoL
Leiden-Padua questionnaire<br>(LEIPAD)<br>[39]	31	QoL<br>*physical functioning*<br>*self-care*<br>*depression and anxiety*<br>*mental functioning*<br>*social functioning*<br>*sexual function*<br>*life satisfaction*	Total score: 0–93<br>Higher scores indicate better QoL
Nottingham Health Profile<br>(NHP)<br>[40]	38	QoL<br>*sleep*<br>*physical activity/mobility*<br>*pain*<br>*energy*<br>*emotional reactions*<br>*social isolation*	Total score: 0–100<br>Higher scores indicate poorer level of health
Satisfaction With Life Scale<br>(SWLS)<br>[41]	5	Life satisfaction	Total score: 5–35<br>Higher scores indicate better QoL

Table S2 *Continued*

Name<br>(Abbreviation, Reference)	Items	Dimension/Subscales	Interpretation
Short Form Health Survey<br>(SF36/20/12)<br>[42]	36//20/12	QoL<br>*physical functioning*<br>*role physical*<br>*bodily pain*<br>*general health*<br>*mental health*<br>*role emotional*<br>*vitality*<br>*social functioning*	Total score: 0–100<br>Higher scores indicate better QoL
St. George's Respiratory Questionnaire<br>(SGRQ)<br>[43]	50	QoL (patients with diseases of airways obstruction)<br>*symptoms*<br>*activity*<br>*impact*	Total score: 0–100<br>Higher scores indicate more limitations
World Health Organization Quality of Life Brief Version<br>(WHOQOL-BREF)<br>[44]	26	QoL<br>*physical health*<br>*psychological health*<br>*social relationships*<br>*environment*	Total score: 16–80<br>Higher scores indicate better QoL
**Physical and psychological functioning (QoL)**			
Multidimensional Fatigue Inventory<br>(MFI)<br>[45]	20	Fatigue<br>*physical fatigue*<br>*reduced activity*<br>*mental fatigue*<br>*reduced motivation*<br>*general fatigue*	Total score: 20–140<br>Higher scores indicate higher degree of fatigue

Table S2 *Continued*

Name<br>(Abbreviation, Reference)	Items	Dimension/Subscales	Interpretation
		**Psychological functioning (QoL)**	
Mental Health Inventory<br>(MHI-18)<br>[46]	18	Mental health<br>*anxiety*<br>*depression*<br>*behavioral control*<br>*positive affect*<br>*general distress*	Total score: 0–100<br>Higher scores indicate better mental health
Perceived Self-Efficacy Scale<br>(PEBS)<br>[47]	10	Self-efficacy	Total score: 5–50<br>Higher scores indicate higher self-efficacy
Rosenberg Self-Esteem Scale<br>(RSES)<br>[48]	10	Self-esteem	Total score: 10–40<br>Higher scores indicate higher self-esteem
Warwick-Edinburgh Mental Well-Being Scale<br>(WEMWBS)<br>[49]	14	Mental well-being	Total score: 14–70<br>Higher scores indicate higher mental well-being
		**Social functioning (QoL)**	
De Jong Gierveld Loneliness Scale<br>(DJGLS)<br>[50]	11	Feelings of loneliness<br>*social loneliness*<br>*emotional loneliness*	Total score: 0–11<br>Higher scores indicate greater loneliness
Inventory of Social Supportive Behaviors<br>(ISSB)<br>[51]	13	Supportive behaviors<br>*behavior*<br>*satisfaction*	Total score: 13–65<br>Higher scores indicate better behavior and satisfaction

Table S2 *Continued*

Name (Abbreviation, Reference)	Items	Dimension/Subscales	Interpretation
Lubben Social Network Scale (LSNS-6) [52]	6	Social network	Total score: 0–30<br>Higher scores indicate larger social networks
Multidimensional Scale of Perceived Social Support (MSPSS) [53]	12	Perceived social support	Total score: 12–84<br>Higher levels of perceived support
Revised Social Support Questionnaire (RSSQ) [54]	6	Social support	Total score: 6–36<br>Higher scores indicate larger social networks
University of California, Los Angeles, Loneliness Scale (UCLA) [55]	20	Social isolation	Total score: 0–93<br>Higher scores indicate higher level of loneli-ness
**Depressive symptoms**			
Beck Depression Inventory (2nd version) (BDI/BDI-II) [56]	21	Depression	Total score: 0–63<br>Higher scores indicate greater depressive symptoms
Center for Epidemiological Stud-ies Depression Scale (CES-D) [57]	20	Depression	Total score: 0–60<br>Higher scores indicate greater depressive symptoms

Table S2 *Continued*

Name<br>(Abbreviation, Reference)	Items	Dimension/Subscales	Interpretation
Depression Anxiety Stress Scale (short version)<br>(DASS21)<br>[58]	21	Depression<br>Anxiety<br>Stress	Total score: 0–42 (3x)<br>Higher scores indicate more psychological distress
Geriatric Depression Scale (Short Form)<br>(GSD/GDS-SF)<br>[59]	30/15	Depression	Total score: 0–15/30<br>Higher scores indicate greater depressive symptoms
Hospital Anxiety and Depression Scale<br>(HADS)<br>[60]	14	Depression<br>Anxiety	Total score: 0–21<br>Higher scores indicate greater depressive symptoms
Profile of Mood States (Short Form)<br>(POMS/POMS-SF)<br>[61]	65/35	Mood states<br>*tension-anxiety*<br>*depression-dejection*<br>*anger-hostility*<br>*vigor-activity*<br>*fatigue-inertia*<br>*confusion-bewilderment*	Higher scores in vigor indicate good mood or emotion, lower scores in the other subscales indicate good mood or emotion
Taiwanese Depression Questionnaire<br>(TDQ)<br>[62]	18	Depression	Total score: 0–54<br>Higher scores indicate greater depressive symptoms
		**Fear of falling (FoF)**	
Activities-Specific Balance Confidence Scale<br>(ABC)<br>[63]	16	Balance confidence	Total score: 0–100<br>Higher scores indicate greater balance confidence

Table S2 *Continued*

Name (Abbreviation, Reference)	Items	Dimension/Subscales	Interpretation
Falls Efficacy Scale (International) (FES/FES-I/Short FES-I) [64]	10/16/7	FoF	Total score: 10–100/16–64/7–28<br>Higher scores indicate greater FoF
Modified Falls Efficacy Scale (MFES) [65]	14	FoF	Total score: 0–10<br>Higher scores indicate greater FoF
Geriatric Fear of Falling Measure (GFFM) [66]	15	FoF<br>*psychosomatic symptoms*<br>*risk prevention*<br>*modifying behavior*	Total score: 0–15<br>Higher scores indicate greater FoF
		**Sleep quality**	
Pittsburgh Sleep Quality Index (PSQI) [67]	19	Sleep quality<br>*subjective sleep quality*<br>*sleep latency*<br>*sleep duration*<br>*habitual sleep efficacy*<br>*sleep disturbances*<br>*sleeping medications*<br>*daytime dysfunction*	Total score: 0–21<br>Higher scores indicate worse sleep quality

Table S3 PEDro scores and sum of the included trials.

Reference	1. Eligibility Specified	2. Subjects Randomly Allocated	3. Concealed Allocation	4. Similar Baseline Values	5. Blinding of Subjects	6. Blinding of Therapist	7. Blinding of Assessor	8. Dropout <15%	9. Received Treatment as Allocated	10. Statistical Between-Group Comparison	11. Point Measures and Variability Provided	Sum (2–11)
[1]	+	+	+	+	–	–	+	+	–	+	+	7
[2]	+	+	+	+	–	–	+	+	+	+	+	8
[3]	+	+	–	+	–	–	+	+	+	+	+	7
[4]	+	+	–	+	–	–	+	–	+	+	+	6
[5]	+	+	–	+	–	–	+	+	–	+	+	6
[6]	+	+	–	+	–	–	–	+	–	+	+	5
[7]	+	+	–	+	–	–	+	+	–	+	+	6
[8]	+	+	–	+	–	–	–	+	–	+	+	5
[9]	+	+	–	+	–	–	–	+	–	+	+	5
[10]	+	+	–	+	–	–	–	+	+	+	+	6
[11]	+	+	+	+	–	–	+	+	+	+	+	8
[12]	+	+	+	+	–	–	+	+	–	+	+	7
[13]	+	+	+	+	–	–	+	+	+	+	+	8

Table S3 *Continued*

Reference	1. Eligibility Specified	2. Subjects Randomly Allocated	3. Concealed Allocation	4. Similar Baseline Values	5. Blinding of Subjects	6. Blinding of Therapist	7. Blinding of Assessor	8. Dropout <15%	9. Received Treatment as Allocated	10. Statistical Between-Group Comparison	11. Point Measures and Variability Provided	Sum (2–11)
[14]	+	+	–	+	–	–	+	+	–	+	+	6
[15]	–	+	–	+	–	–	–	+	–	+	+	5
[16]	+	+	–	+	–	–	+	+	+	+	+	7
[17]	+	+	–	+	–	–	–	+	+	+	+	6
[18]	+	+	–	+	–	–	–	+	+	+	+	6
[19]	+	+	+	+	–	–	+	+	+	+	+	8
[20]	+	+	+	+	–	–	–	+	+	+	+	7
[21]	+	+	–	+	–	–	–	+	–	+	+	5
[22]	+	+	–	+	–	–	+	–	+	+	+	6
[23]	+	+	+	+	–	–	+	+	+	+	+	8
[24]	+	+	–	+	–	–	+	+	+	+	+	7
[25]	+	+	+	+	–	–	–	+	–	+	+	6
[26]	+	+	–	+	–	–	–	+	–	+	+	5

Table S3 *Continued*

Reference	1. Eligibility Specified	2. Subjects Randomly Allocated	3. Concealed Allocation	4. Similar Baseline Values	5. Blinding of Subjects	6. Blinding of Therapist	7. Blinding of Assessor	8. Dropout <15%	9. Received Treatment as Allocated	10. Statistical Between-Group Comparison	11. Point Measures and Variability Provided	Sum (2–11)
[27]	+	+	–	+	–	–	–	+	–	+	+	5
[28]	+	+	+	+	–	–	+	+	+	+	+	8
[29]	+	+	+	+	–	–	–	+	–	+	+	6
[30]	+	+	–	+	–	–	+	+	–	+	+	6
[31]	+	+	+	+	–	–	+	+	+	+	+	8
[32]	+	+	+	+	–	–	–	+	+	+	+	7
[33]	+	+	–	+	–	–	+	+	+	+	+	7
[34]	+	+	+	+	–	–	+	+	+	+	+	8
[35]	+	+	–	+	–	–	–	+	–	+	+	5
[36]	+	+	–	+	–	–	–	+	–	+	+	5
[37]	+	+	+	+	–	–	+	+	+	+	+	8

Note. PEDro: Physiotherapy Evidence Database scale; +: yes; –: no.

References

1. Aibar-Almazán, A.; Hita-Contreras, F.; Cruz-Díaz, D.; de la Torre-Cruz, M.; Jiménez-García, J.D.; Martínez-Amat, A. Effects of Pilates training on sleep quality, anxiety, depression and fatigue in postmenopausal women: A randomized controlled trial. *Maturitas* **2019**, *124*, 62–67, doi:10.1016/j.maturitas.2019.03.019.
2. Aibar-Almazán, A.; Martínez-Amat, A.; Cruz-Díaz, D.; De la Torre-Cruz, M.J.; Jiménez-García, J.D.; Zagalaz-Anula, N.; Pérez-Herrezuelo, I.; Hita-Contreras, F. Effects of Pilates on fall risk factors in community-dwelling elderly women: A randomized, controlled trial. *Eur. J. Sport Sci.* **2019**, *19*, 1386–1394, doi:10.1080/17461391.2019.1595739.
3. Alp, A.; Cansever, Ş.; Görgeç, N.; Yurtkuran, M.; Topsaç, T. Effects of Tai Chi exercise on functional and life quality assessments in senile osteoporosis. *Turkiye Klin. J. Med. Sci.* **2009**, *29*, 687–695.
4. Chan, A.W.K.; Lee, A.; Lee, D.T.F.; Sit, J.W.H.; Chair, S.Y. Evaluation of the sustaining effects of Tai Chi Qigong in the sixth month in promoting psychosocial health in COPD patients: A single-blind, randomized controlled trial. *Sci. World J.* **2013**, doi:10.1155/2013/425082.
5. Chan, A.W.K.; Yu, D.S.F.; Choi, K.C. Effects of Tai Chi qQgong on psychosocial well-being among hidden elderly, using elderly neighborhood volunteer approach: A pilot randomized controlled trial. *Clin. Interv. Aging* **2017**, *12*, 85–96, doi:10.2147/CIA.S124604.
6. Chen, K.M.; Chen, M.H.; Chao, H.C.; Hung, H.M.; Lin, H.S.; Li, C.H. Sleep quality, depression state, and health status of older adults after silver Yoga exercises: Cluster randomized trial. *Int. J. Nurs. Stud.* **2009**, *46*, 154–163, doi:10.1016/j.ijnurstu.2008.09.005.
7. Chyu, M.C.; James, C.R.; Sawyer, S.F.; Brismée, J.M.; Xu, K.T.; Poklikuha, G.; Dunn, D.M.; Shen, C.L. Effects of Tai Chi exercise on posturography, gait, physical function and quality of life in postmenopausal women with osteopaenia: A randomized clinical study. *Clin. Rehabil.* **2010**, *24*, 1080–1090, doi:10.1177/0269215510375902.
8. Dong, L.; Lee, J.-B.; Kim, Y.-K.; Kim, Y.-S. The effects of health Qigong training of elderly single women on pain consciousness and depression. *Int. J. Appl. Sport. Sci.* **2013**, *25*, 118–126, doi:10.24985/ijass.2013.25.2.118.
9. Fakhari, M. Effects of Tai Chi exercise on depression in older adults: A randomized controlled trial. *Bali Med. J.* **2017**, *6*, 679, doi:10.15562/bmj.v6i3.706.
10. Fan, B.; Song, W.; Zhang, J.; Er, Y.; Xie, B.; Zhang, H.; Liao, Y.; Wang, C.; Hu, X.; Mcintyre, R.; et al. The efficacy of mind-body (Baduanjin) exercise on self-reported sleep quality and quality of life in elderly subjects with sleep disturbances: A randomized controlled trial. *Sleep Breath.* **2020**, doi:10.1007/s11325-019-01999-w.
11. Fransen, M.; Nairn, L.; Winstanley, J.; Lam, P.; Edmonds, J. Physical activity for osteoarthritis management: A randomized controlled clinical trial evaluating hydrotherapy or Tai Chi classes. *Arthritis Care Res. (Hoboken).* **2007**, *57*, 407–414, doi:10.1002/art.22621.
12. Gabizon, H.; Press, Y.; Volkov, I.; Melzer, I. The effects of Pilates training on balance control and self-reported health status in community-dwelling older adults: A randomized controlled trial. *J. Aging Phys. Act.* **2016**, *24*, 376–383, doi:10.1123/japa.2014-0298.
13. Greendale, G.A.; Huang, M.-H.; Karlamangla, A.S.; Seeger, L.; Crawford, S. Yoga decreases kyphosis in senior women and men with adult onset hyperkyphosis: Results of a randomized controlled trial. *J. Am. Geriatr. Soc.* **2009**, *57*, 1569–1579, doi:10.1111/j.1532-5415.2009.02391.x.
14. Groessl, E.J.; Maiya, M.; Schmalzl, L.; Wing, D.; Jeste, D. V. Yoga to prevent mobility limitations in older adults: Feasibility of a randomized controlled trial. *BMC Geriatr.* **2018**, *18*, doi:10.1186/s12877-018-0988-8.
15. Hosseini, H.; Esfirizi, M.F.; Marandi, S.M.; Rezaei, A. The effect of Tai Chi exercise on the sleep quality of the elderly residents in Isfahan, Sadeghieh elderly home. *Iran. J. Nurs. Midwifery Res.* **2011**, *16*, 55–60.
16. Hosseini, L.; Kargozar, E.; Sharifi, F.; Negarandeh, R.; Memari, A.H.; Navab, E. Tai Chi Chuan can improve balance and reduce fear of falling in community dwelling older adults: A randomized control trial. *J. Exerc. Rehabil.* **2018**, *14*, 1024–1031, doi:10.12965/jer.1836488.244.
17. Hsu, C.Y.; Moyle, W.; Cooke, M.; Jones, C. Seated Tai Chi versus usual activities in older people using wheelchairs: A randomized controlled trial. *Complement. Ther. Med.* **2016**, *24*, 1–6, doi:10.1016/j.ctim.2015.11.006.
18. Hsu, C.Y.; Moyle, W.; Cooke, M.; Jones, C. Seated T'ai Chi in older Taiwanese people using wheelchairs: A randomized controlled trial investigating mood states and self-efficacy. *J. Altern. Complement. Med.* **2016**, *22*, 990–996, doi:10.1089/acm.2015.0191.
19. Huang, T.T.; Yang, L.H.; Liu, C.Y. Reducing the fear of falling among community-dwelling elderly adults through cognitive-behavioural strategies and intense Tai Chi exercise: A randomized controlled trial. *J. Adv. Nurs.* **2011**, *67*, 961–971, doi:10.1111/j.1365-2648.2010.05553.x.

20. Irwin, M.R.; Olmstead, R.; Motivala, S.J. Improving sleep quality in older adults with moderate sleep complaints: A randomized controlled trial of Tai Chi Chih. *Sleep* **2008**, *31*, 1001–1008, doi:10.5665/sleep/31.7.1001.
21. Li, F.; Harmer, P.; McAuley, E.; John Fisher, K.; Duncan, T.E.; Duncan, S.C. Tai Chi, self-efficacy, and physical function in the elderly. *Prev. Sci.* **2001**, *2*, 229–239, doi:10.1023/A:1013614200329.
22. Lipsitz, L.A.; Macklin, E.A.; Travison, T.G.; Manor, B.; Gagnon, P.; Tsai, T.; Aizpurua, I.I.; Lo, O.-Y.; Wayne, P.M. A cluster randomized trial of Tai Chi vs. health education in subsidized housing: The MI-WiSH study. *J. Am. Geriatr. Soc.* **2019**, *67*, 1812–1819, doi:10.1111/jgs.15986.
23. Logghe, I.H.J.; Zeeuwe, P.E.M.; Verhagen, A.P.; Wijnen-Sponselee, R.M.T.; Willemsen, S.P.; Bierma-Zeinstra, S.M.A.; van Rossum, E.; Faber, M.J.; Koes, B.W. Lack of effect of Tai Chi Chuan in preventing falls in elderly people living at home: A randomized clinical trial. *J. Am. Geriatr. Soc.* **2009**, *57*, 70–75, doi:10.1111/j.1532-5415.2008.02064.x.
24. Lü, J.; Huang, L.; Wu, X.; Fu, W.; Liu, Y. Effect of Tai Ji Quan training on self-reported sleep quality in elderly Chinese women with knee osteoarthritis: A randomized controlled trail. *Sleep Med.* **2017**, *33*, 70–75, doi:10.1016/j.sleep.2016.12.024.
25. Mortazavi, H.; Tabatabaeicher, M.; Golestani, A.; Armat, M.; Yousefi, M. The effect of Tai Chi exercise on the risk and fear of falling in older adults: A randomized clinical trial. *Mater. Socio Medica* **2018**, *30*, 38, doi:10.5455/msm.2018.30.38-42.
26. Nguyen, M.H.; Kruse, A. A randomized controlled trial of Tai chi for balance, sleep quality and cognitive performance in elderly Vietnamese. *Clin. Interv. Aging* **2012**, *7*, 185–189, doi:10.2147/CIA.S32600.
27. Nick, N.; Petramfar, P.; Ghodsbin, F.; Keshavarzi, S.; Jahanbin, I. The effect of Yoga on balance and fear of falling in older adults. *PM R* **2016**, *8*, 145–151, doi:10.1016/j.pmrj.2015.06.442.
28. Noradechanunt, C.; Worsley, A.; Groeller, H. Thai Yoga improves physical function and well-being in older adults: A randomised controlled trial. *J. Sci. Med. Sport* **2017**, *20*, 494–501, doi:10.1016/j.jsams.2016.10.007.
29. Oh, C.; Kang, H. Effects of Tai Chi exercise on the body composition, self-efficacy and life satisfaction of older adults in Korean local community. *Int. J. Gerontol.* **2019**, *13*, 134–138, doi:10.6890/IJGE.201906_13(2).0007.
30. Oken, B.S.; Zajdel, D.; Kishiyama, S.; Flegal, K.; Dehen, C.; Haas, M.; Kraemer, D.F.; Lawrence, J.; Leyva, J. Randomized, controlled, six-month trial of Yoga in healthy seniors: Effects on cognition and quality of life. *Altern. Ther. Health Med.* **2006**, *12*, 40–47.
31. Tajik, A.; Rejeh, N.; Heravi-Karimooi, M.; Kia, P.S.; Tadrisi, S.D.; Watts, T.E.; Griffiths, P.; Vaismoradi, M. The effect of Tai Chi on quality of life in male older people: A randomized controlled clinical trial. *Complement. Ther. Clin. Pract.* **2018**, *33*, 191–196, doi:10.1016/j.ctcp.2018.10.009.
32. Teut, M.; Knilli, J.; Daus, D.; Roll, S.; Witt, C.M. Qigong or Yoga versus no intervention in older adults with chronic low back pain—A randomized controlled trial. *J. Pain* **2016**, *17*, 796–805, doi:10.1016/j.jpain.2016.03.003.
33. Tew, G.A.; Howsam, J.; Hardy, M.; Bissell, L. Adapted yoga to improve physical function and health-related quality of life in physically-inactive older adults: a randomised controlled pilot trial. *BMC Geriatr.* **2017**, *17*, doi:10.1186/s12877-017-0520-6.
34. Tiedemann, A.; O'Rourke, S.; Sesto, R.; Sherrington, C. A 12-week Iyengar Yoga program improved balance and mobility in older community-dwelling people: A pilot randomized controlled trial. *J. Gerontol. Ser. A Biol. Sci. Med. Sci.* **2013**, *68*, 1068–1075, doi:10.1093/gerona/glt087.
35. Tsang, H.W.H.; Lee, J.L.C.; Au, D.W.H.; Wong, K.K.W.; Lai, K.W. Developing and testing the effectiveness of a novel health qigong for frail elders in Hong Kong: A preliminary study. *Evid. based Complement. Altern. Med.* **2013**, *2013*, 827392, doi:10.1155/2013/827392.
36. Wang, D.S. Feasibility of a Yoga intervention for enhancing the mental well-being and physical functioning of older adults living in the community. *Act. Adapt. Aging* **2010**, *34*, 85–97, doi:10.1080/01924781003773559.
37. Zhao, Y.; Chung, P.K.; Tong, T.K. Effectiveness of a community-based exercise program on balance performance and fear of falling in older nonfallers at risk for falling: A randomized, controlled study. *J. Aging Phys. Act.* **2016**, *24*, 516–524, doi:10.1123/japa.2015-0224.
38. Herdman, M.; Gudex, C.; Lloyd, A.; Janssen, M.; Kind, P.; Parkin, D.; Bonsel, G.; Badia, X. Development and preliminary testing of the new five-level version of EQ-5D (EQ-5D-5L). *Qual. Life Res.* **2011**, *20*, 1727–1736, doi:10.1007/s11136-011-9903-x.
39. De Leo, D.; Diekstra, R.F.W.; Lonnqvist, J.; Lonnqvist, J.; Cleiren, M.H.P.D.; Frisoni, G.B.; Buono, M. Dello; Haltunen, A.; Zucchetto, M.; Rozzini, R.; et al. Leipad, an internationally applicable instrument to assess quality of life in the elderly. *Behav. Med.* **1998**, *24*, 17–27, doi:10.1080/08964289809596377.

40. Hunt, S.M.; McEwen, J.; McKenna, S.P. Measuring health status: A new tool for clinicians and epidemiologists. *J. R. Coll. Gen. Pract.* **1985**, *35*, 185–188.
41. Diener, E.; Emmons, R.A.; Larsen, R.J.; Griffin, S. The satisfaction with life scale. *J. Pers. Assess.* **1985**, *49*, 71–75, doi:10.1207/s15327752jpa4901_13.
42. Ware, J.E.; Sherbourne, C.D. The MOS 36-item short-form health survey (SF-36). *Med. Care* **1992**, *30*, 473–483, doi:10.1097/00005650-199206000-00002.
43. Jones, P.W.; Quirk, F.H.; Baveystock, C.M. The St. George's respiratory questionnaire. *Respir. Med.* **1991**, *85*, 25–31, doi:10.1016/S0954-6111(06)80166-6.
44. Skevington, S.M.; Lotfy, M.; O'Connell, K.A. The World Health Organization's WHOQOL-BREF quality of life assessment: Psychometric properties and results of the international field trial a report from the WHOQOL Group. *Qual. Life Res.* **2004**, *13*, 299–310, doi: 10.1023/B:QURE.0000018486.91360.00.
45. Smets, E.M.A.; Garssen, B.; Bonke, B.; de Haes, J. The multidimensional fatigue inventory (MFI) psychometric qualities of an instrument to assess fatigue. *J. Psychosom. Res.* **1995**, *39*, 315–325, doi:10.1016/0022-3999(94)00125-O.
46. Veit, C.T.; Ware, J.E. The structure of psychological distress and well-being in general populations. *J. Consult. Clin. Psychol.* **1983**, *51*, 730–742, doi:10.1037/0022-006X.51.5.730.
47. Riggs, M.L.; Knight, P.A. The impact of perceived group success-failure on motivational beliefs and attitudes: A causal model. *J. Appl. Psychol.* **1994**, *79*, 755–766, doi:10.1037/0021-9010.79.5.755.
48. Rosenberg, M. Self esteem and the adolescent self-image. *Science.* **1965**, *148,* 804, doi:10.1126/science.148.3671.804.
49. Tennant, R.; Hiller, L.; Fishwick, R.; Platt, S.; Joseph, S.; Weich, S.; Parkinson, J.; Secker, J.; Stewart-Brown, S. The Warwick-Dinburgh mental well-being scale (WEMWBS): Development and UK validation. *Health Qual. Life Outcomes* **2007**, *5*, 63, doi:10.1186/1477-7525-5-63.
50. Gierveld, J.D.J.; Van Tilburg, T. A 6-item scale for overall, emotional, and social loneliness: Confirmatory tests on survey data. *Res. Aging* **2006**, *28*, 582–598, doi:10.1177/0164027506289723.
51. Barrera, M.; Sandler, I.N.; Ramsay, T.B. Preliminary development of a scale of social support: Studies on college students. *Am. J. Community Psychol.* **1981**, *9*, 435–447, doi:10.1007/BF00918174.
52. Lubben, J.E. Assessing social networks among elderly populations. *Fam. Community Heal. J. Heal. Promot. Maint.* **1988**, *11*, 42–52, doi:10.1097/00003727-198811000-00008.
53. Zimet, G.D.; Dahlem, N.W.; Zimet, S.G.; Gordon, K.; Farley, G.K. The multidimensional scale of perceived social support. *J. Pers. Assess.* **1988**, *52*, 30–41, doi:10.1207/s15327752jpa5201_2.
54. Sarason, I.G.; Sarason, B.R.; Shearin, E.N.; Pierce, G.R. A brief measure of social support: Practical and theoretical implications. *J. Soc. Pers. Relat.* **1987**, *4*, 497–510, doi:10.1177/0265407587044007.
55. Hughes, M.E.; Waite, L.J.; Hawkley, L.C.; Cacioppo, J.T. A short scale for measuring loneliness in large surveys: Results from two population-based studies. *Res. Aging* **2004**, *26*, 655–672, doi:10.1177/0164027504268574.
56. Beck, A.; Ward, C.; Mendelson, H.; Mock, M.; Erbaugh, J. An inventory for measuring depression. *Arch. Gen. Psychiatry* **1961**, *4*, 561–571, doi:10.1001/archpsyc.1961.01710120031004.
57. Andresen, E.M.; Malmgren, J.A.; Carter, W.B.; Patrick, D.L. Screening for depression in well older adults: Evaluation of a short form of the CES-D. *Am. J. Prev. Med.* **1994**, *10*, 77–84, doi:10.1016/s0749-3797(18)30622-6.
58. Lovibond, P.F.; Lovibond, S.H. The structure of negative emotional states: Comparison of the depression anxiety stress scales (DASS) with the beck depression and anxiety inventories. *Behav. Res. Ther.* **1995**, *33*, 335–343, doi:10.1016/0005-7967(94)00075-U.
59. Yesavage, J.A.; Brink, T.L.; Rose, T.L.; Lum, O.; Huang, V.; Adey, M.; Leirer, V.O. Development and validation of a geriatric depression screening scale: A preliminary report. *J. Psychiatr. Res.* **1982**, *17*, 37–49, doi:10.1016/0022-3956(82)90033-4.
60. Zigmond, A.S.; Snalth, R.P. The hospital anxiety and depression scale. *Acta Psychiatr. Scand.* **1983**, *67*, 361–370, doi:10.1093/occmed/kqu024.
61. McNair, D.M.; Lorr, M. An analysis of mood in neurotics. *J. Abnorm. Soc. Psychol.* **1964**, *69*, 620–627, doi:10.1037/h0040902.
62. Lee, Y.; Yang, M.J.; Lai, T.J.; Chiu, N.M.; Chau, T.T. Development of the Taiwanese depression questionnaire. *Chang Gung Med. J.* **2000**, *23*, 688–694.
63. Powell, L.; Myers, A. The Activities-specific Balance Confidence (ABC) Scale. *J. Gerontol.* **1995**, *50A*, 28–34, doi: 10.1093/gerona/50a.1.m28.

64. Kempen, G.; Yardley, L.; van Haastregt, J.; Zijlstra, G.; Beyer, N.; Hauer, K.; Todd, C. The short FES-I: a shortened version of the falls efficacy scale-international to assess fear of falling. *Age Ageing* **2008**, *37*, 45–50, doi:10.1093/ageing/afm157.
65. Tinetti, M.E.; Richman, D.; Powell, L. Falls efficacy as a measure of fear of falling. *J. Gerontol.* **1990**, *45*, 239–243, doi:10.1093/geronj/45.6.P239.
66. Huang, T.T. Geriatric fear of falling measure: Development and psychometric testing. *Int. J. Nurs. Stud.* **2006**, *43*, 357–365, doi:10.1016/j.ijnurstu.2005.04.006.
67. Buysse, D.J.; Reynolds, C.F.; Monk, T.H.; Berman, S.R.; Kupfer, D.J. The Pittsburgh sleep quality index: A new instrument for psychiatric practice and research. *Psychiatry Res.* **1989**, *28*, 193–213, doi:10.1016/0165-1781(89)90047-4.
